# Dissecting the Heterogeneous Population Genetic Structure of *Candida albicans*: Limitations and Constraints of the Multilocus Sequence Typing Scheme

**DOI:** 10.3389/fmicb.2019.01052

**Published:** 2019-05-10

**Authors:** Marina Muñoz, Luz Maira Wintaco, Shirly Alexandra Muñoz, Juan David Ramírez

**Affiliations:** ^1^Grupo de Investigaciones Microbiológicas – UR (GIMUR), Programa de Biología, Facultad de Ciencias Naturales y Matemáticas, Universidad del Rosario, Bogotá, Colombia; ^2^Centro de Tecnología en Salud (CETESA), Upqua SAS, Bogotá, Colombia; ^3^Programa de Doctorado en Ciencias Biomédicas y Biológicas, Universidad del Rosario, Bogotá, Colombia; ^4^Unidad de Salud de Ibagué (USI) E.S.E, Ibagué, Colombia

**Keywords:** *Candida albicans*, multilocus sequence typing, molecular epidemiology, population genetic structure, recombination

## Abstract

*Candida albicans* is a fungal opportunistic pathogen of significant public health importance mainly due to the recent emergence of strains with increased aggressiveness and antifungal resistance. Here, we aimed to describe the epidemiological profiles and approximate the population structure of *C. albicans* by analyzing the *C. albicans* multilocus sequence typing (MLST) database (Calb-MLST-DB), which contains the largest publically available dataset for this species. Based on 4,318 database isolates, we confirmed the ubiquitous nature of *C. albicans* including a group of diploid sequence types (DSTs) obtained from Healthy individuals exclusively (taken as an indicator of lack of association with illnesses in its host), until isolates established from Non-Healthy individuals (potentially associated with pathogenic processes) and other DSTs reported in both types (Healthy and Non-Healthy). The highest number of reported DSTs was related to blood, oral and vaginal swabs (32.4, 20.5, and 13.8%, respectively). High genetic diversity was observed in the seven housekeeping genes included in the MLST scheme, with a diverse population structure (154 clonal complexes, CCs; and a high number of singletons, *n* = 1,074). Phylogenetic reconstruction on the concatenated alignment of these housekeeping genes for all the reported DSTs (*n* = 3,483) was partially concordant with the CC assignment, however, an absence of bootstrap threshold supported nodes or p-distance, and the lack of association with the other epidemiological variables, evidenced the limitations of the MLST scheme. Marked genetic admixture signals were identified by STRUCTURE, with the majority being attributable to recombination events according to the RDP program results, although another type of exchange event cannot be ruled out. Our results reaffirm the genetic diversity inherent in the genes used for the MLST scheme, which are associated with the chromosomal remodeling already proposed for *C. albicans*. This was also corroborated with an internal validation at a micro geographical scale. Despite these results are biased due to the unavailability of considering the broad global spectrum of *C. albicans* isolates around the world. This suggests that the strategy used to population type this pathogen should be reevaluated to improve epidemiological monitoring of its health impact.

## Introduction

*Candida albicans*, a ubiquitous eukaryotic yeast, can colonize humans, other animals and a wide range of ecological niches (Hall and Noverr, 2017). Although commensalism (not involving physiological interactions or dependency) is the most common interaction in healthy individuals, some factors contribute to the interaction becoming pathogenic, whereby it can exploit the host in a detrimental way (Dheilly et al., 2015). In humans, the proliferation of *C. albicans* is associated with a wide spectrum of diseases (from urogenital to deep-seated infections, mainly in hospitalized patients), which cause high morbidity and mortality, making it the most common opportunistic fungal pathogen of public health importance over the last two decades (Poulain, 2015).

The study of the genetic diversity in *Candida* species has allowed researchers to identify associations with severe infections, and for that reason, different typing methods have been employed to describe the epidemiological profiles for those species (Garcia-Hermoso et al., 2016). Multilocus sequence typing (MLST) was proposed as a high-resolution method based on nucleotide polymorphisms in multiple housekeeping genes, which allow the identification of the unique diploid sequence types (DSTs) (Tomasini et al., 2014), that can be even used in medically important fungal species (Alanio et al., 2017). A consensus MLST scheme for *C. albicans*, based on the following housekeeping genes, was selected: aspartate aminotransferase (AAT1a), acetyl-CoA carboxylase (ACC1), ATP-dependent permease (ADP1), mannose phosphate isomerase (MPIb), alanyl-RNA synthetase (SYA1), vacuolar protein sorting (VPS13), and glucose-6-phosphate dehydrogenase (ZWF1b)(Bougnoux et al., 2003, 2004).

MLST data have been produced in different studies worldwide and are centralized in public databases. The PubMLST database website^1^ is currently recognized as the most complete repository for MLST data (Jolley and Maiden, 2010). One of the PubMLST databases is *C. albicans* MLST database (“Calb-MLST-DB”^2^). Besides being a repository for MLST sequences and profiles, PubMLST databases contain epidemiological information from reported clinical isolates. MLST data have also been employed to develop multilocus sequence analysis (MLSA), a feature directed at deciphering diversity, genetic population structure and reconstructing evolutionary relationships between closely related strains in different microbial species (Tomasini et al., 2014). Consequently, in 2014, a genetic population structure was proposed for *C. albicans*. It was based on 17 major clades identified via clusters from the phylogenetic reconstruction of MLST data from a set of 1,391 isolates, using a nucleotide polymorphism proportion (P-distance) of 0.04 for minor clades and 0.07 for mayor clades as the threshold (McManus and Coleman, 2014). These clusters are consistent with the geographical location information and are grouped in clonal complexes, a clustering strategy considered to be the consensus (McManus and Coleman, 2014).

After the definition of a consensus MLST scheme for *C. albicans* (Bougnoux et al., 2003), a genetic population structure predominantly clonal was initially propose for this fungal opportunistic pathogen (Tavanti et al., 2005). However, the wide phenotypical polymorphism in *C. albicans* was subsequently supported by its high genomic diversity (Benedetti et al., 2016; Wang et al., 2018). This fungal pathogen has a 32 Mb diploid genome comprising eight pairs of chromosomes with a high degree of heterozygosity (Hirakawa et al., 2015). The subsequent studies have confirmed a genome plasticity: recombination, gross chromosomal rearrangements, gene replacement and cryptic mating (Selmecki et al., 2010). Some signals of evolutionary molecular events in *C. albicans* are evident by the presence of polymorphisms, copy number variations, gene inversions, subtelomeric hypervariation, loss of heterozygosity and whole or partial chromosome aneuploidies in its genome (McManus and Coleman, 2014; Hirakawa et al., 2015). Currently, molecular epidemiology studies have allowed researchers to type a high number of *C. albicans* strains, and subsequently identify a large number of DSTs, many of which could not be assigned to a clade. Thus, implementing a strategy that allows the *C. albicans* genetic population structure to be evaluated is now a necessity for this research field. Although, currently there are next generation sequencing techniques that could provide more complete information about circulating isolates in different environments and hosts, they still represent a challenge for different laboratories due to the high costs of sequencing, the restriction in developing countries, the complexity of data analysis, among others. Therefore, the MLST is still considered as a cost-effective strategy for the typing and epidemiological monitoring of some species (Page et al., 2017).

Given the utility of the MLST for the typing of clinical isolates, the description of the population structure, and the availability of public MLST data for *C. albicans* in the global repositories, particularly the Calb-MLST-DB, the aims of this study were (i) to describe the epidemiological profiles of clinical isolates deposited in such databases, (ii) to identify the molecular features (DNA and Insertion-Deletion polymorphisms, InDels; evolutionary divergence; degree of linkage disequilibrium and recombination signals) of the housekeeping genes included in the *C. albicans* MLST scheme, and (iii) to evaluate the intra-taxa diversity from all the sequences deposited. The MLSA conducted here provides a platform for depicting the updated genetic population structure of *C. albicans* and to explore the evolutionary dynamics of this fungal pathogen.

## Materials and Methods

### Data Retrieval

The MLST sequences used in this study were downloaded from the *Candida albicans* MLST Databases (Calb-MLST-DB) website^3^. This data source is a curated and publicity available repository which uses two linked databases. The first one, “isolates database,” contains a repository of epidemiological information a collection of isolates that represent the total known diversity of *C. albicans*^4^. According to the information described in Calb-MLST-DB, the isolates database contained information about 4,318 isolates when our study was performed (date of update: 16-03-2017). The second database is a repository for sequences and profile definitions^5^ where the MLST profiles and allele sequences of 3,483 DSTs are reported (date of update: 16-01-2018). Inferences about the *C. albicans* genetic population structure were made from the complete set of data, however, because various strains and data sources submitted by laboratories to Calb-MLST-DB could be heterogeneous, a micro-geographic analysis was conducted as an alternative representative source of high-quality data. Under this premise, the isolates from United Kingdom were selected due to: (i) it corresponds to one of the countries with the highest number of isolates reported in the Calb-MLST-DB (Supplementary Figure 1), (ii) the geographical and sample collection are fairly standard (which was taken as an indicator that they were systematically collected), (iii) correspond to the country where the MLST strategy analysis approach was implemented as a tool for the study of population genetics and evolution of microbial species (Tirosh and Barkai, 2011) and iv) historically, a greater number of molecular epidemiology works have been developed based on the MLST strategy in a wide range of hosts (Reuter et al., 2016; Zou et al., 2018).

### Description of the Epidemiological Profiles

The epidemiological information was exported from the isolate database in a tab-delimited text file, which contained information about the isolate collection (geographical location, year) and its host (source: human or non-human animal, and in the case of isolates obtained from a human host, some reports included the age, sex and diagnosis). Descriptive statistical analyses were conducted over the complete set of variables. The year of isolate establishment and host age were considered as quantitative variables and their means and corresponding standard deviations were recorded. The other variables, including the identified DSTs, were considered as categorical variables, and they were expressed in terms of frequencies and proportions. The number of reports for each variable was considered to be the total number of data for each statistical analysis. The distribution of quantitative variables is reported in terms of percentiles. The association between categorical variables was evaluated using Chi^2^-tests (χ^2^). All analyzes were performed in the statistical program STATA 12^®^, setting the significance level at 5% for all the hypothesis tests.

### Analyzing Housekeeping Genes

Molecular Evolutionary Genetics Analysis software, version 7 (MEGA7), was used for determining the nucleotide composition of the alleles reported for each molecular marker (Kumar et al., 2016), including variable sites (being those positions containing at least two different nucleotides between the set of reported alleles) and parsimony-informative sites (variable sites containing at least two or more nucleotide types, where two or more occur with a minimum frequency of two). Microsoft Excel was used for calculating the parsimony informative sites per number of variable sites rate to facilitate comparable data among the marker diversity.

The utility of *C. albicans* MLST scheme was determined through the calculation of Typing Efficiency (TE) and Discrimination power (DP) for seven housekeeping genes using MLSTest software version 1.0.1.23 (Tomasini et al., 2013). Additionally, a scheme optimization analysis was developed to identify the optimal number of loci and the best combination required to find the higher number of DSTs.

The molecular features of each housekeeping gene included in the *C. albicans* MLST scheme were evaluated using DnaSP v5 software (Librado and Rozas, 2009). Different parameters were evaluated for each housekeeping gene: (i) DNA polymorphisms, measured by the total number of mutations (Eta); number of polymorphic/segregating sites, (S); average number of nucleotide differences (k); number of haplotypes (h); haplotype diversity (Hd, at the genetic polymorphism level and its distribution); nucleotide diversity (π, as the average number of nucleotide differences per site between two sequences and its sampling variance); and the theta (θ) index per site, calculated as 4Nμ (for an autosomal gene from a diploid organism), with N being the effective population size and μ the number of polymorphic (segregating) sites. Each index reported here has its corresponding standard deviation, (ii) InDel polymorphisms, considering the total number of InDel sites and the average length per event; the number of InDel haplotypes; InDel haplotype diversity; InDel diversity per site; and θ index (per sequence) from the total number of InDel events (θ_InDel_), and (iii) evolutionary divergence tests: Tajima’s test estimation (computed as the difference between the mean number of pairwise differences and the number of segregating sites, based on the neutral model prediction) was performed using DnaSP v5 software (Librado and Rozas, 2009), and the ratios of non-synonymous to synonymous substitutions (dN/dS) (as an estimation of average codon-based evolutionary divergence) were calculated using the Fixed Effects Likelihood (FEL) tool (Zhang et al., 1998), which is available in the Datamonkey database (Delport et al., 2010) for each housekeeping gene.

Non-random associations between the nucleotide variants at different polymorphic sites were evaluated for each housekeeping gene by calculating the degree of linkage disequilibrium (LD) for all polymorphic sites using DnaSP software v5 (Librado and Rozas, 2009). The following parameters were calculated to quantify LD: D, D’, and R. The LD results were plotted against nucleotide distance and their absolute value was then used to estimate the relationship between LD and physical distance through regression analyses (|D|, |D’| and Rˆ2). For |D’| values, a second regression equation was calculated excluding the |D’| = 1 (+1 and −1) values. LD confidence intervals for the whole data were determined by coalescent-based simulations and considered to be signals of intragenic recombination. Associations among the nucleotide variants were calculated by computing B and Q statistics (Rozas et al., 2003).

### Predicting Clonal Complexes (CCs)

A second file containing the allelic profiles of all the DSTs reported for *C. albicans* (*n* = 3,484) was downloaded from the sequence database^6^. Relatedness between the DSTs was inferred by identifying differences between the allelic profiles using the optimized version of the eBURST algorithm (goeBURST-1.2.1), which allows the identification of groups (defined as CCs) where the members have alleles in common with another member of the defined group. The geoBURST algorithm, which provides information about patterns of descent under conditions comparable to those for the majority of natural microbial populations, was also used (Francisco et al., 2009).

### Phylogenetic Reconstructions

The concatenated sequences for the total number of DSTs reported in Calb-MLST-DB (*n* = 3,492) were downloaded from the locus/sequence definitions database. The sequences were aligned using the PyNAST method, which is a python implementation based on the NAST algorithm, and was selected because it allows best-matching of sets of sequences with high identity (minimum percent identity, 75%) (Caporaso et al., 2010). The concatenated sequences for homologous housekeeping genes included in the *C. albicans* MLST scheme for three different *Candida* strains were included in the alignments. The first one was *C. albicans* strain SC5314, which corresponds to a clinical isolate belonging to the predominant clade representing almost 40% of all the isolates worldwide. The second reference sequence included was *C. africana* ATCC MYA-2669, a species closely related to *C. albicans*, which has recently been described (Romeo and Criseo, 2011). A sequence from *C. dubliniensis*, the closest relative of *C. albicans* (McManus and Coleman, 2014), was used as outgroup (access number: Cd36_08460). Housekeeping gene alignments were obtained by delimiting the regions from the concatenated alignment for all the DSTs.

Phylogenetic reconstructions for the concatenated alignment were developed using two methods: (i) RAxML (Randomized Axelerated Maximum Likelihood) version 8, which is reported to be the most accurate maximum-likelihood method for intensive searching of topology space (Stamatakis, 2014), and (ii) FastTree version 2.1. 9, which is a fast and accurate method for inferring approximate maximum-likelihood phylogenetic trees, with high performance on large alignments (Price et al., 2010). GTRGAMMA and Jukes-Cantor were the evolution models used for the RAxML and FastTree methods, respectively, because they were predicted to be the models with the best-balanced support considering the CAT approximation. To implement the FastTree method, a preliminary step to exclude heterozygosity sites was conducted. The initial topology was refined and optimized by including different rounds of comparisons by log-likelihoods for nearest-neighbor interchange “NNIs” branch swapping (minimum evolution- NNIs “ME-NNIs” and maximum-likelihood-NNIs “ML-NNIs”) and a minimum-evolution subtree-pruning-regrafting “ME-SPRs”). A final round of the ML-NNIs was implemented to turn off the heuristics and generate the final topology to optimize all the lengths (Price et al., 2010). The method with the clearest grouping results for the concatenated sequence was used to obtain trees for each housekeeping gene included in the scheme. The bootstrap (BT) method with 1,000 replicates was used to estimate the variance in the phylogenetic reconstructions (taking 99.9% as the threshold).

### Identifying Recombination Signals

Different tests to identify molecular rearrangements (nucleotide loss and duplication events, hybridization, horizontal gene transfer or recombination) were developed to detect the molecular signals that could help to elucidate the *C. albicans* genetic population structure. The first test conducted was SplitsTree (Huson and Bryant, 2006), which employs the neighbor-net method. A further analysis to identify the minimum number of recombination events per housekeeping gene was conducted using DnaSP software v5 (Librado and Rozas, 2009).

Genetic admixture signals were predicted using STRUCTURE software 2.3.4. (Hubisz et al., 2009), considering *K* = 18 as the number of populations, because this is the number of clades previously described for *C. albicans*. The analysis was based on 600,000 iterations of the Markov chain Monte Carlo (MCMC) algorithm following a burn-in of 60,000 iterations. Allocating DSTs to populations was represented through a triangle plot, with the aim of identifying the number of clusters in the positions where they belong, being the result associated with congruence in the predicted classification. Additionally, a tree plot was generated to represent the genetic distances among the 18 K clusters of classically proposed populations, which enables both population splits and migration events to be captured (Pickrell and Pritchard, 2012).

Finally, to confirm and characterize the existence of recombination events, an additional analysis was conducted in the Recombination Detection Program version 4 (RDP4) (Martin et al., 2015), taking as input the alignment of the concatenated sequences for all the DSTs reported. This software makes use of different methods such as RDP software, whereas the others traditionally used are GENECONV, BootScan, Maximum Chi Square (MaxiChi), Chimera, Sister Scanning (SiScan), 3Seq, the VisRD method, and BURT) (Martin et al., 2005). In combination, these methods allow the use of RDP, thereby representing the best available strategy to describe recombination events, while also detecting the signals attributable to a process other than recombination.

## Results

### Epidemiological Profiles

The epidemiological information from 4,318 isolates in total, as described at the last update (03-16-2017), was exported using the script available at https://pubmlst.org/bigsdb?page=plugin&name=Export&db=pubmlst_calbicans_isolates//pubmlst.org/bigsdb?page=plugin&name=Export&db=pubmlst_calbicans_isolates. After preliminary verification, it was found that the isolate reporting frequency for each DST was from 1 (for most isolates) to 123 (for DST-69), whereas no information for 227 DSTs was found. Therefore, a dataset with 4,552 entries was established for the epidemiological analyses. Although this dataset does not represent the population sample because the information from different studies is voluntarily deposited in Calb-MLST-DB, it does represent the total known diversity for *C. albicans*, making it a scientifically valuable dataset.

The typing information allowed us to determine that the majority of the DSTs have been reported once (60.7%) or twice (10.3%) in the Calb-MLST-DB, with the maximum reporting frequency being 2.7% for DST 69 (*n* = 123), followed very closely by DST 79 at 1.4% (*n* = 66). The other DSTs have a frequency below 1%. A group of 1,489 isolates had useful information for analyzing the genetic population structure, including a clade proposed according to the traditional classification (1–18) and the clonal complex determined by the eBURST algorithm (from 1 to 98, in addition to singletons). This number of reports is equivalent to 32.7% of all the isolates.

The entries with geographical origin information allowed us to determine that the DSTs deposited in Calb-MLST-DB at the update date come from 56 countries, with the most frequent origin being China (22.2%), followed by the United Kingdom (15.3%), United States (8.4%), and France (6.6%). No source information was available for 5.1% of the isolates. The reports from other countries represented less than 5% frequency each. The distribution of the reports for isolates by country can be reviewed in Supplementary Figure 1.

In our analysis of the reporting frequency per year, data from a set of 2,739 isolates were identified, which was established as being 1954–2016, with the average year of establishment for the isolates being 2006 (with a SD: 7.2 years). Most isolates were established after 2012 (percentile 75%). The number of isolates reported in Calb-MLST-DB and United Kingdom per year is described in Supplementary Figure 2.

A set of 3,323 isolates had information about the host from which they were obtained. Fifteen different sample sources were identified, including a set of 135 isolates of non-human animal origin, with manatees (22.2%; *n* = 30) and starlings (11.9%; *n* = 16) being the most frequent origin within this group. The other isolate sources of non-human animal origin were < 10% of all the reports. In the case of human isolates, the following was found: (i) 626 isolates with age information had an age range of 0–98 years (Mean = 52.6 years; SD = 24.7); (ii) most of the data with host sex information (*n* = 1,039) were obtained from women (59.1%, *n* = 614); and (iii) the isolation sources in the hosts were varied, the most frequent being blood (32.4%, *n* = 1,026), followed by oral (20.5%; *n* = 650) and vaginal swabs (13.8%; *n* = 437). The other sample sources did not exceed a 10% reporting frequency for each. In the case of micro-geographic analyses (United Kingdom), all isolates were obtained from humans, being predominantly isolated from blood and oral swab, with 46.7% (*n* = 261) and 34.1 (*n* = 190), respectively.

**FIGURE 1:**
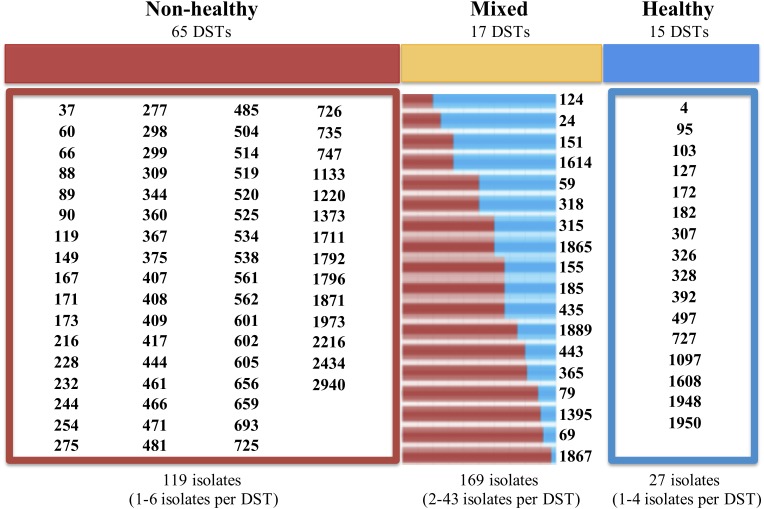
Distribution of DSTs according to their potential impact on the hosts. “Non-Healthy” correspond to the DSTs identified in the isolates obtained from individuals with some pathology. “Healthy” are defined as DSTs reported from the isolates obtained from healthy individuals. The “Mixed” category included the isolations obtained from individuals with pathologies, as well as from healthy. The numbers at the bottom of each panel describe the total number of DSTs reported in each category, and the frequency range for the reported DSTs is shown in parentheses. In the case of “Mixed” DSTs, the bars represent the frequency of Non-Healthy (in red) and commensal (blue) isolates reported for each case.

**FIGURE 2:**
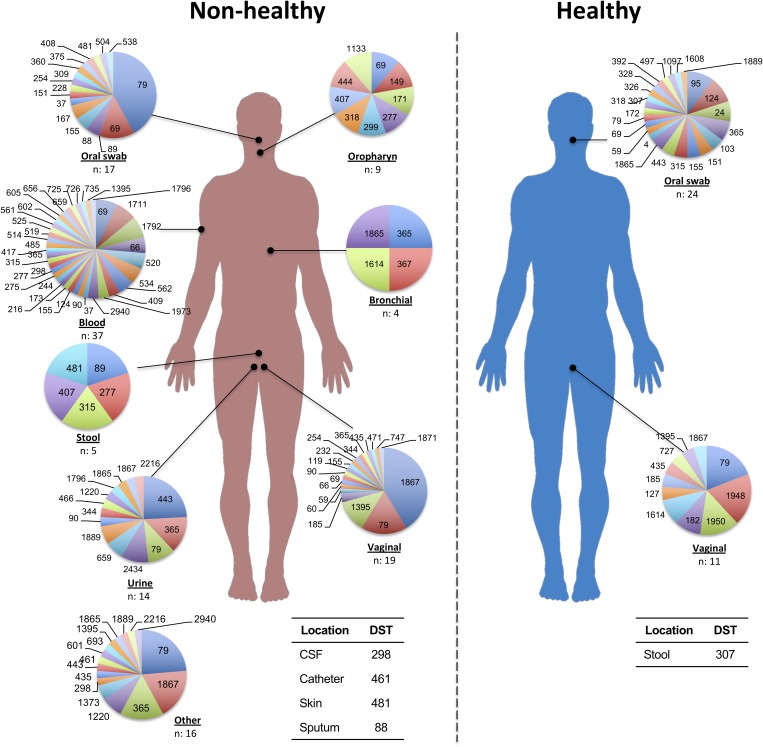
DSTs reported by sample source with potential to be Healthy or Non-Healthy. Each circle represents the total number of isolations reported for the main sample sources (with the corresponding *n*-value shown in the lower part). Each DST frequency was calculated by considering the total number of DSTs per source.

Additionally, we identified a set of 992 reports with diagnostic information, which formed the basis for the classification according to their potential impact on the host. Isolates with frequencies of 1 or 2 (over the whole database) were excluded from subsequent analyzes, thus generating a set of 315 isolates with 3 or more reports that were used for analyzing their potential impact on hosts. Isolates that were reported as being exclusively from healthy individuals (*n* = 27; 8.6%) were cataloged as “Healthy,” while those obtained only from patients with some pathology, were defined as “Non-Healthy” (*n* = 119; 37.8%). Among the most frequent pathologies were vaginitis (9.0%, *n* = 89) and AIDS (6.0%; *n* = 60), whereas the others showed a reporting frequency of less than 3.0%. Any remaining isolates that were present in both the Healthy and Non-Healthy categories were included in a third group defined as “Mixed” (*n* = 169; 53.7%). The description of the DSTs reported in each host type (Healthy, Non-Healthy or Mixed) is provided in Figure 1. Additionally, we analyzed the frequency of reporting for the different DSTs by sample source, discriminating between Healthy and Non-Healthy, and found that high numbers of DSTs defined as Non-Healthy were present across a wide range of infection sources, with the greatest DST diversity in blood (*n* = 37 DSTs) and vaginal (*n* = 19 DSTs) infections. In the case where the DSTs were present as Commensals, only three sample sources were identified: oral swabs (*n* = 24 DSTs), vaginal swabs (*n* = 11 DSTs), and feces (*n* = 1 DST). The DST distribution by sample source is shown in Figure 2. The micro geographic analysis revealed that only 177 isolates from the United Kingdom had information on the host status from which they were isolated, of which 30.5% corresponded to healthy volunteers (*n* = 54), while the remaining 69.5% were categorized as Non-Healthy. A total of 15 and 30 DSTs were identified, respectively.

### Genetic Features of the Housekeeping Gene Markers

The utility of seven housekeeping genes was calculated using MLSTest software, over the complete dataset exclusively. The results for DP were from 0.887 for MPIb to 0.953 for AAT1 (Supplementary Figure 3A). For the case of TE the results were extremely high for all housekeeping genes, with data from 1,964 for SYA1 to 5,436 for VPS13 (Supplementary Figure 3A). Scheme optimization analysis showed that although the optimum number of loci required identifying the largest number of DSTs was 7, where a combination of genes allows identifying 3,482 DSTs, from two housekeeping could be identified more than one thousand DTSs (Supplementary Figure 3B). The analysis of different molecular marker combinations showed that in all cases VPS13 was present in those with the best performance to define high number of DSTs (Supplementary Figure 3C), this fact is in agreement with its high TE and DP results.

These analyzes were conducted on the sequences from all the alleles reported for each gene^7^. The first analysis phase allowed us to ascertain that the number of alleles reported for each gene was between 113 (for ACC1) and 302 (for VPS13). With the DNA polymorphisms, the gene with the highest Eta and S (which are measures of the average number of differences) was ADP1 (*n* = 68 and 64, respectively), while the less polymorphic gene was AAT1a (Eta, 41; S, 36). Our calculation of k showed that MPIb and ADP1 genes scored the highest (5.02513 and 4.87723, respectively) of the genes we analyzed. In most cases Hd was very close to 1 (>0.9504), whereas the results for π were smaller and more heterogeneous, with values ranging from 0.00868 for ZWF1b to 0.00598 for ACC1. The theta index was from 0.01474 (for ZWF1) to 0.02290 (for MPIb) in all cases.

Although preliminary analyzes of the molecular characteristics of the housekeeping genes, measured in terms of DNA polymorphisms, did not show large differences between the markers, the InDel polymorphism analysis showed that only three of the genes present had evidence of this type of molecular rearrangement; they were AAT1a, ACC1, and SYA1, the latter having the largest number of InDel sites (*n* = 26) with the average length per event being high (13,000). However, a negative result for the parameter of evolutionary divergence, Tajima’s D, was found for all housekeeping genes (below -1.25716). Although the results are not statistically significant (*P* > 0.10), we observed that they were all <0. Therefore, we can infer that rare, high frequency alleles are present. The dN/dS ratio were between 0.065, for the case of ZWF1, and 1.239 for VPS14 (only gene with a ratio >1). Two alleles had to be excluded from the alignment used for the analysis of dN/dS ratio of SYA, because their difference in sequence markedly impacted the identity of the alignment, going from 55.7% for the 236 alleles included in all the analyzes, to 76.4% eliminating those two alleles (*n* = 234), and did not allow the calculation of this rate. The housekeeping gene analysis results are shown in the Table 1.

**Table 1:** Genetic features of the housekeeping gene markers.

	Parameter	Housekeeping gene						
		AAT1a	ACC1	ADP1	MPIb	SYA1	VPS13	ZWF1b
	Reported alleles	178	113	170	174	236	302	292
	Length	373	407	443	375	391	403	491
	Total number of sites analyzed	376	408	443	375	404	403	491
DNA polymorphisms	Total number of sites (excluding gaps/missing data)	370	406	379	375	327	403	491
	Eta	41	44	68	60	55	58	54
	S	36	39	64	55	50	54	50
	k	3.2735	3.2735	4.87723	5.02513	3.76073	3.62833	4.26427
	h	77	62	107	106	109	150	143
	hd [SD]	0.964 [0.003]	0.954 [0.005]	0.9504 [0.0073]	0.9632 [0.0048]	0.9648 [0.0034]	0.9739 [0.0020]	0.9734 [0.0027]
	π	0.00885	0.00598	0.01101	0.0134	0.00995	0.009	0.00868
	θ	0.01512 [0.00385]	0.01607 [0.00420]	0.02275 [0.00529]	0.02290 [0.00542]	0.01975 [0.00461]	0.01926 [0.00434]	0.01474 [0.00338]
Insertion-Deletion (InDel) polymorphisms	Total number of InDel sites	6	2	0	0	26	0	0
	Average length per event	1.004	1.000	–	–	13.000	–	–
	Number of InDel haplotypes	3	2	–	–	2	–	–
	InDel hd	0.023	0.018	–	–	0.009	–	–
	InDel π	0.023	0.00009	–	–	0.00004	–	–
	θ_InDel_	0.777	0.335	–	–	0.299	–	–
Evolutionary divergence	Tajima’s *D*-test	−1.35675	−1.35675	−1.59297	−1.4344	−1.52285	−1.56658	−1.25716
	dN/dS	0.300	0.341	0.249	0.506	0.154^∗^	1.230	0.065

*Eta, total number of mutations; S, number of polymorphic sites; k, average number of nucleotide differences; h, number of haplotypes; hd, haplotype diversity; π, nucleotide diversity; θ, Theta index; dN/dS, non-synonymous/synonymous rate. ^∗^ 234 alleles included in the analysis.*

The LD analysis allowed us to determine that the genes with the largest number of polymorphic sites, possibly corresponding to recombination events, were ADP1 (with 61 sites), and MPIb and VPS13 (both with 51 sites), and with the LD indexes, we found that most of them were close to 0.0000 or negative with the exception of AAT1 (Supplementary Table 1).

### CC Identification

**FIGURE 3:**
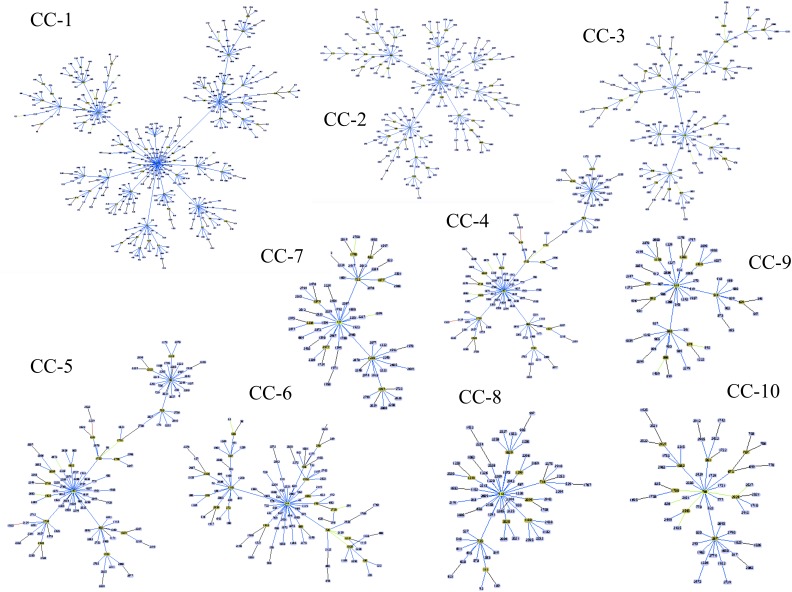
The population structure at the genetic level for *C. albicans* according to the eBURST algorithm (Francisco et al., 2009). The DSTs included in the 10 main Clonal Complexes (CCs) are shown, with the following being the predicted founder of each CC (in ascending order): DST-69; -124; -155; -365; -344; -461; 601; -727; -918, and -538. The extended results of the eBURST algorithm are shown in Supplementary Table 2.

Our analysis based on the BURST algorithm for the 3,483 allelic profiles reported in the sequence database showed that the genetic population of *C. albicans* was definable as a set of 154 CCs, taking six identical loci as the criteria. CC-1, the most representative CC, includes 13.9% (*n* = 483) of all the reported DSTs, with DTS-69 being its predicted founder. A group of 10 CCs (with CC-1 included) collected together 43.3% of the DSTs (*n* = 1,509 DSTs). The other CCs each have a frequency lower than 2.0% (Supplementary Table 2). Interestingly, about one third of the reported DSTs (30.8%; *n* = 1,074) were reported as singletons. The main CCs are graphically depicted in Figure 3. In the United Kingdom dataset, the analysis of population structure based on CC, allowed to identify 24 CCs, including the CC-1 in a total of 77 isolates (Supplementary Figure 4 and Supplementary Table 3).

### Phylogenetic Relationships

**FIGURE 4:**
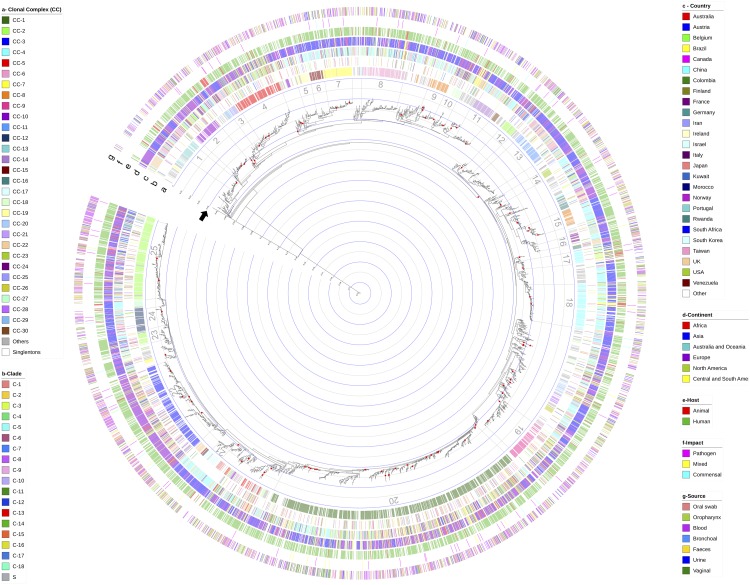
Phylogenetic reconstruction based on the alignment of the concatenated sequences for the total number of reported DSTs (*n* = 3,493). The FastTree method was implemented, considering the Jukes-Cantor evolution model (identified as the best-balanced support by the CAT approximation). The red points on the nodes represent well-supported BTs (≥99.9). The concentric circles inside the tree indicate the p-distance. The black arrow indicates a p-distance of 0.04 and represents the threshold reported for the definition of minor clades (McManus and Coleman, 2014). Each external circle represents the comparison using the different evaluated variables, being (a) clonal complex (CC); (b) classically defined clades; (c) country where the isolations were established; (d) grouping by continent from where the isolates were obtained, and the host type: humans and non-human animals; (f) the potential impact on the hosts: Non-Healthy, commensals or mixed; and (g) sample source from where the isolates were obtained. Bars in the external circles indicate the existence of data for each isolate. Color was assigned according to the categories described by the conventions described on the sides of the tree.

Phylogenetic reconstructions based on all the concatenated sequences available in the Calb-MLST-DB, plus the dataset for the three reference sequences described in the methodology section (total *n* = 3,485), showed a lack of resolution using the RAxML method, which generated a uniform topology with all the DSTs grouped in a single cluster with poor support (Supplementary Figure 5), while the phylogenetic tree generated using the FastTree method (Figure 4) allowed us to identify a group of DSTs with very high support in terms of the BT; hence, this method was selected for the other phylogenetic analyzes.

When considering the cut-off point for the analysis (BT ≥ 99.9%), 125 clusters were identifiable. With the reference sequences, we found that *C. dubliniensis* Cd36_08460 allowed for rooting of the tree and *C. albicans* strain SC5314 was located within one of the identified clusters, an expected result in both cases. However, we also found that *C. africana* ATCC MYA-2669 grouped within one of the detected clusters.

A clustering analysis based on the strategy classically used for *C. albicans* and considering the p-distance cut-off for classification in major (0.07) and minor (0.04) clades, was conducted on the phylogenetic tree obtained from the concatenated sequences for all the DSTs reported. Complexity in the tree topology was apparent, and there was a lack of concordance with the grouping based on the BT values, which represented the main challenges for defining the groups that allowed us to identify a single “major clade” and there was a greater number of “minor clades” compared with the clades previously reported (McManus and Coleman, 2014). The p-distance delineation in the phylogenetic tree is shown in the concentric circles inside Figure 4. Two findings were of interest from this analysis: (i) there are clusters with p-distances between 0.04 and 0.07 (which may or may not have BT support), which are grouped independently (*n* = 4), and (ii) a level of additional clustering was observed in some of the “minor clades” identified, with a p-distance less than 0.04 but with BT support, with one example being the “minor clade” defined by us as No. 21 in Figure 3, in which are included about half of the analyzed DSTs (*n* = 1,526), and where at least 30 internal clusters can be identified, using BT as the parameter.

The DST distribution in the phylogenetic tree was evaluated by considering different variables. First, the assignment of CC (the only variable with the outcome based on the MLST scheme), is shown in external circle a in Figure 4, where the distribution of the 30 CCs with the highest number of DSTs reported revealed a clustering associated with the clusters identified in the phylogenetic tree. Comparing these two variables allowed the identification of at least 25 clusters, which although also lacking BT support, represent the only grouping level found in the analyzed dataset. Interestingly, distributed throughout the phylogenetic tree are DSTs included in the category “Others,” which correspond to rare CCs (where < 10 DSTs are included) and singletons (S).

The allocation of previously reported clades and the main epidemiological characteristics reported in the isolate database (whose analyzes were previously described in this section as epidemiological profiles) were contrasted against the phylogenetic tree we obtained. However, no grouping profiles for the DSTs were found according to any of the variables evaluated (country, continent, host, source and potential impact, as represented in the external circles of Figure 3 in that order, with the inner circle as the country).

The topology of the phylogenetic trees generated from the individual housekeeping genes alignment for the complete set of isolates, showed that all the DSTs grouped in a single cluster (with a p-distance < 0.04) and most, with the exception of ADP1 and ZWF1b, revealed a global absence of BT values that exceed the established cut-off point (Supplementary Figure 6). The phylogenetic reconstruction at micro geographical scale showed a close relationship among isolates reported from United Kingdom, with p-distance less than < 0.01 for the most cases (concentric circle inside Supplementary Figure 7). Despite the high number of isolates (*n* = 550) and corresponding DSTs (*n* = 405), none of the clusters revealed reached a p-distance 0.04, which could suggest that all DSTs would belong to the same Clade.

### Recombination Signatures

The phylogenetic networks analysis based on the neighbor-net algorithm (using the SplitsTree package), allowed us to identify a close relationship between almost all the reported DSTs, with clear evidence of cross-linking events, which could represent an initial signal for genetic material exchange events between them. A single exception was identified, where DST-2 (reported in China and Spain), showed a clear divergence from all other DSTs. The results of the SplitsTree analysis can be reviewed in Supplementary Figure 8. This analysis was also performed at the microgeographic level (United Kingdom isolates), showing the existence of cross-linking events even in the data set considered to be more homogeneous (Supplementary Figure 9).

**FIGURE 5:**
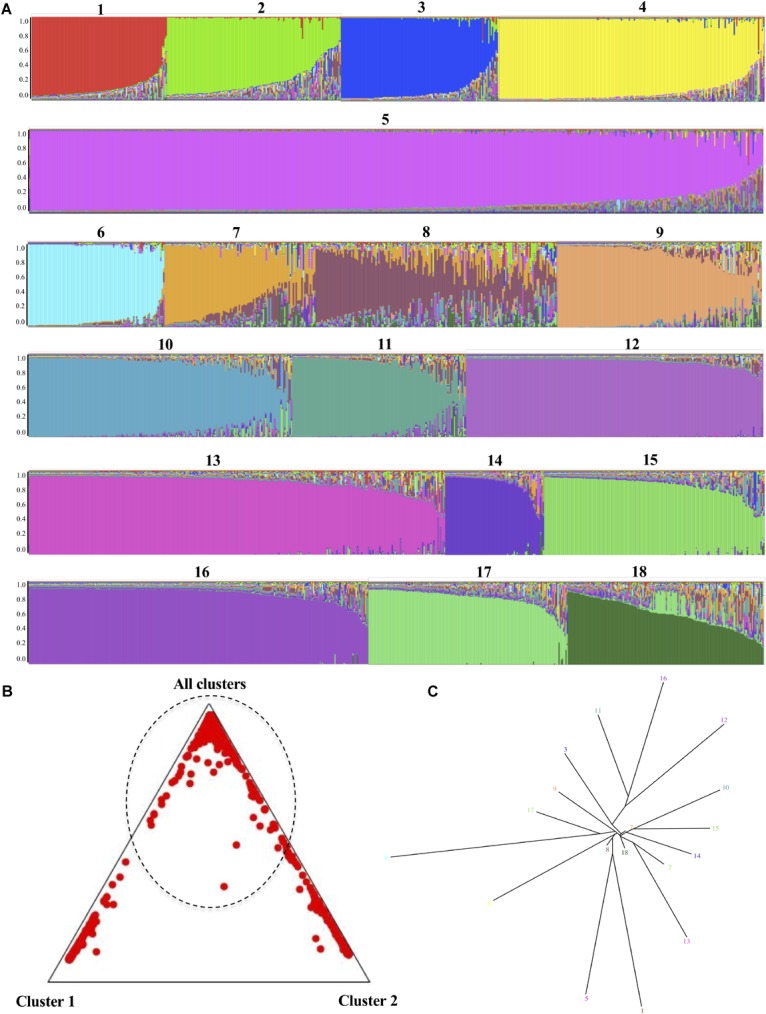
Identification of genetic admixture signals in *C. albicans* using STRUCTURE software 2.3.4 (Hubisz et al., 2009). The analysis parameters under consideration were the number of clades previously described for this species, which is included as the number of populations (*K* = 18), together with 600,000 iterations of the Markov chain Monte Carlo algorithm following a burn-in of 60,000 iterations. **(A)** Bar plot sorted by Q for *K* = 18. The presence of more than one color in each bar represents genetic admixture signals. **(B)** Triangle plot that shows the distribution of the DSTs in clusters; that is, either 1, 2 or all others, the latter category including all those whose probability of grouping cannot be defined. **(C)** Tree plot representing the genetic distance between the K structure clusters.

The second analysis aimed at identifying recombination was developed in STRUCTURE, where K, the number of major clades previously accepted for intra-taxa consensus classification (*n* = 18), showed clear signals of genetic material exchange events observed between the pre-established populations (Figure 5A). Although some of the populations showed a relatively stable structure at the genetic level, such as K 5 and 12, others show how in samples 7 and 8 an exchange of material could well be diverted to introgressions or recombination events. The triangle plot generated for assigning individuals to populations showed that more than half of the analyzed DSTs could not be assigned to a cluster (dotted lines in Figure 5B), because they have a homogeneous probability of belonging to any cluster. The evaluation of the genetic distance among the K structure clusters, represented in the tree plot (Figure 5C), allowed us to ascertain that the populations defined as 7, 8, and 18, and even 2, are in the central zone of the plot, with a loss of nucleotide distance, which could be an indicator of the existence of individuals that are migrants or admixed. The STRUCTURE analysis results did not allow us to infer the presence of defined populations. Although the information regarding the Clades/populations was limited for the analysis at the microgeographic level, it was possible to identify signs of the existence of migrants or admixed even at this level (Supplementary Figure 8).

The biological relevance of the possible presence of recombination events in this fungal pathogen prompted us to perform an additional test to identify and characterize such events, using RDP4 software. The results showed evidence of seven genetic material exchange events among the DSTs, five of which were identified as potential recombinants, however, most events are possibly attributable to a process not involving recombination (Supplementary Table 4).

## Discussion

Typing strategies represent the baseline to identify and monitor potentially pathogenic strains, and contribute to the implementation of control measures for the affected individuals to prevent the spread of infection (Sabat et al., 2013). The MLST scheme for *C. albicans* that was proposed in 2003 (Bougnoux et al., 2003) has made it possible to advance in the characterization of populations of this fungal pathogen (McManus and Coleman, 2014) and make preliminary proposals about its population structure at the genetic level (McManus and Coleman, 2014), however, no molecular epidemiology analysis of the isolates by this type of analysis has been done at a global scale. For these reasons, this study aimed to describe the epidemiological profiles, analyze the molecular characteristics, and make an approximation of the population structure of a set of *C. albicans* isolates typed using the *C. albicans* MLST scheme globally approved (Bougnoux et al., 2003, 2004) and submitted to PubMLST databases (the most complete dataset that currently exists and that represents the total diversity known for this fungal pathogen) (Jolley and Maiden, 2010).

When we dissected the MLST database, at the epidemiological profile level, we found that *C. albicans* can be considered an innocuous microorganism in different regions of the world (at least 56 countries, but with an over representation in five countries: China, United Kingdom, United States and France), in human and animal hosts, and can be present either in healthy individuals, or in non-healthy, but additionally some of the identified DSTs have the capacity to establish both biological relationships (Healthy or Non-Healthy), which we defined in this work as “mixed” (Figure 1). This last group includes the isolates with higher reporting frequencies in the database (53.7%), in comparison with Non-Healthy (37.8%) or Healthy (8.6%) DSTs. These findings concord with the information available specifically for *C. albicans*, where its cosmopolitan character has been widely reported (Mayer et al., 2013), but also align with evolutionary theories that support an increase in individuals/strains that can adapt to diverse environments (West et al., 2006). Furthermore, it should be noted that the small number of isolates with exclusively commensal profiles that we identified could result from the design of the epidemiological studies in that the data reported to Calb-MLST-DB are mostly for isolates from individuals that present some degree of pathology (Figure 2).

Regarding the analyzes we conducted on the PubMLST sequences, a preliminary analysis of the diversity of any of the microorganisms for which data is included could be aimed at comparing the number of reported DSTs vs. the number of isolates from which they were established. In the case of *C. albicans*, the 3,483 DSTs reported at the time our investigations were conducted were established using the 4,318 isolates described in the database (representing a rate of 0.81). These findings agree with previous reports about phylogeny and evolution of *Candida albicans* based on *C. albicans* MLST scheme, where the DST reporting rate was 0.85 (McManus and Coleman, 2014), and they are higher than what was found for related fungal pathogens, such as *C. glabrata*, for the 77 DSTs reported for 231 isolations, which reach a rate of 0.33^8^. This preliminary indicator, together with the frequency of new DTSs being identified each time an isolate is reported in the database, is established as the first evidence for genetic diversity in this fungal pathogen and the potential drawbacks of the MLST as it stands.

An evaluation of the molecular characteristics of the seven housekeeping genes was conducted to identify the molecular basis in support of the typing scheme (Table 1). Preliminary analyzes were directed at identifying DNA polymorphisms, and the ACC1 and AAT1a genes were found to be the most conserved of the test genes, showing the lowest number of alleles, the lowest results for Eta, S, and k, as well as the lowest diversity indexes at both the nucleotide and haplotype levels. In contrast, the ADP1 and MPIb genes were the most diverse of the tested genes at the nucleotide level. The second part of the analysis showed that although most of the genes did not contain InDel polymorphisms, interestingly, AAT1 and ACC1, which are more conserved at the nucleotide level, contained 6 and 2 InDel sites, which showed InDel hd and a π value of < 0.02. However, during this phase of the analysis the most remarkable result was for SYA1, which despite showing average DNA polymorphism results (for the set of housekeeping genes analyzed), revealed the signs of 26 InDel events, which despite showing low InDel hd and π (<0.09) values were considerably longer than for the first two genes described (13,000 vs. 1,000). Our analysis of the molecular characteristics of the seven housekeeping genes was completed by the identification of evolutionary divergence signals, in which the presence of rare alleles at high frequencies (by Tajima’s D test results under -1) and purifying selection signals were (by dN/dS results, <0.5) were found for most of the genes, except for SYA1, where the presence of rare alleles (227 and 231) could be associated to horizontal gene transfer (Tavanti et al., 2005), and VPS13, where their result of dN/dS rate >1.0 makes it a candidate gene to be experiencing positive selection (Yang and Bielawski, 2000).

In general, the findings for the seven housekeeping genes allowed us to infer that they have different selection profiles. These findings are relevant to the use of the MLST scheme, due to both of the discrimination power (which allows researchers to differentiate individuals belonging to different groups) such as the typing efficiency (as an indicator of the grouping of members with common characteristics), define how researchers report on the implementation of MLST schemes in bacterial species (Varni et al., 2014) and eukaryotic microbial pathogens (Tomasini et al., 2014). However, during the design of the *C. albicans* MLST scheme, only the discrimination power was considered, being selected the minimum set of fragments that allowed differentiation as independent DSTs for all the evaluated isolates (Bougnoux et al., 2003), and the importance of typing efficiency had not yet been recognized. That premise affect considerably the performance of the MLST scheme at the clinical level, because it would not be possible the molecular tracking on disease outbreaks, for which it is possible to find commonality points between nearby strains. In this way, it is clear that under the base of the current MLST scheme is not possible to propose an optimal typing method for *C. albicans* from this combination of housekeeping genes, and it is necessary to consider the following points:

The implementation of molecular typing schemes should consider the grouping of individuals with similar characteristics. This hypothesis has been previously proposed as part of the utility of the MLST schemes for the description of molecular epidemiology, population genetics, and pathogenic roles of, for example, *Helicobacter pylori* (Suzuki et al., 2012) and *Staphylococcus aureus* (Huh and Chung, 2016), and even for eukaryotic pathogens such as *Pneumocystis pneumonia* (De Boer et al., 2011). Thus, it has been suggested that “to assign isolates to ‘types’ must clearly provide enough information to distinguish one ‘type’ from another, but without being discriminatory that every isolate is unique” (Turner and Feil, 2007). This is also supported when the description of the population structure at the genetic level using the BURST algorithm revealed that the *C. albicans* isolates could be grouped into 125 CCs with a high number of singletons, revealing again the exaggerated discriminatory power of this strategy (Figure 3). This is a clear challenge for the accurate molecular epidemiology of *C. albicans*, and the advent of genomic data reinforces the need to propose a more suitable and less resolutive MLST scheme for this fungal pathogen.

The phylogenetic analyzes based on the concatenated sequence of the seven housekeeping genes (Figure 4), revealed an inconsistency in the identification of clusters/clades (considering the criteria P-distance threshold, according to the parameters used for this purpose in smaller populations (McManus and Coleman, 2014), or BT, considering the consensus statistical support for phylogenetic trees) (Deng et al., 2013). These findings could be related to the use of the concatenated sequence of the seven housekeeping genes, although such an approach has been useful for describing evolutionary pathways in bacterial species (Maiden, 2006; Munoz et al., 2017) and in some pathogenic fungi such as the *Cryptococcus c*omplex members (Munoz et al., 2018), but it was not informative with this combination of markers in *C. albicans*. This could be related to the molecular characteristics of the genes, which are exposed to different evolutionary processes (as described in the previous sections) and that biologically do not represent a single set of analyzes. The different molecular characteristics exhibited by the genes may be influenced by their locations at the chromosomal level, because genes such as ACC1 (the less variable gene), which is located on chromosome 3, are historically described as lacking rearrangements, including repetitive sequences (Chindamporn et al., 1998), whereas genes with greater variability are found on chromosome 1 (ADP1 and ZWF1) and are associated with the generation of variants displaying trisomy (44). Chromosome 6 (SYA1) is also described as highly unstable, even presenting with unusual sizes in some strains (Rustchenko, 2007). These findings present additional evidence about the limits to which the MLST scheme can be used for the purpose of analyzing a population structure.

Although 10 years ago the use of this type of approach (based on the MLST scheme evaluated here) was useful for assigning isolations to the proposed clades (Odds et al., 2007) and the phylogenetic relationships inferred seemed to reveal a predominantly clonal structure (Tavanti et al., 2005). Increases in the number of isolates analyzed have abolished these hypotheses. Among the main findings that contribute to these premises include the identification of hybrid strains or introgressed genomic regions resulting from genetic exchange (Forche et al., 2008), which represent the primary evidence of the existence of rearrangements at the molecular level in *C. albicans*. A more complete picture about the origin of diversity among populations of this fungal pathogen has begun to emerge thanks to the development of studies at the whole genome level, which have revealed chromosome instability in this fungal pathogen (Rustchenko, 2007). A finding supported by a ploidy plasticity considered to be a rapid and reversible strategy for adaptation to adverse conditions (Berman, 2016). Furthermore, the high frequency of extensive heterozygosity between chromosome homologs and gene family expansions, when occurring together, could favor pathogenicity in particular *C. albicans* strains. Other alternative sources for the excessive diversity found in some of the molecular markers could be the high frequency of heterozygosity in *C. albicans*, and the fixation of rearrangements as gene inversions and subtelomeric hypervariation, which are favored by the plasticity of the genome and which are frequent in other eukaryotes such as *Leishmania* (Ubeda et al., 2014) and *Trypanosoma* (Berna et al., 2018). It should be noted that these types of mechanisms are considered atypical in fungal species, but they have been reported as frequent strategies associated with improving the fitness of this type of species (Storchova, 2014). Thus, it is necessary to design better strategies that allow detailed characterization of the origin of this type of molecular rearrangement.

Generally, the high genetic diversity indices found in the housekeeping genes that are part of the MLST scheme (Table 1), the heterogeneous population structure that is inferred from the set of DSTs reported in PubMLST databases (Figure 3), the restrictions in inferring phylogenetic relationships among them (Figure 4), and the strong signs of structural remodeling (Figure 5), provide evidence for the possible existence of alterations in the probability of replication of certain populations (loss of panmixia) (Waples and Gaggiotti, 2006), which could be one of the mechanisms used to fix these molecular characteristics.

Finally, the results of this work allow us to confirm the useful of MLST approach to deciphering the wide range of epidemiological profiles in which *C. albicans* can be identified. One of the limitation of this study is that various strains and data sources submitted by laboratories to the database make it difficult to explain the representative of *C. albicans* population. However, this is the only publicly available repository of metadata and DNA sequences to conduct this type of analysis. Nevertheless, the heterogeneous features of the molecular markers included in the consensus MLST scheme for this microorganism, together with the genetic diversity of this microorganism, have revealed several limitations that lead to propose properly that this approach might not be an adequate tool to describe *C. albicans* population dynamics. Collectively, this allow us to propose that the design and implementation of an efficient typing scheme (by means of MLST and/or whole genome sequencing) for *C. albicans* still represents a paradigm that requires prompt adjustment to favor the implementation of epidemiological studies that are equipped to contribute to infection control in this potentially pathogenic fungus.

## Author Contributions

MM and JR led the project and analyzed the results. MM, LW, and SM conducted the epidemiological and phylogenetic analyzes. MM and SM wrote the manuscript. JR revised the final version. All authors reviewed and approved the final manuscript.

## Conflict of Interest Statement

The authors declare that the research was conducted in the absence of any commercial or financial relationships that could be construed as a potential conflict of interest.

## Footnotes

1 https://pubmlst.org/

2 https://pubmlst.org/calbicans/

3 https://pubmlst.org/calbicans/

4 https://pubmlst.org/bigsdb?db=pubmlst_calbicans_isolates

5 https://pubmlst.org/bigsdb?db=pubmlst_calbicans_seqdef

6 https://pubmlst.org/bigsdb?db=pubmlst_calbicans_seqdef&page=downloadProfiles&scheme_id=1

7 https://pubmlst.org/bigsdb?db=pubmlst_calbicans_seqdef&page=downloadAlleles

8 https://pubmlst.org/cglabrata/

## Supplementary Material

FIGURE S1 Distribution of the reported isolates by country.

FIGURE S2 The number of isolates reported per year for **(A)** total of reported isolates; and **(B)** isolates from United Kingdom.

FIGURE S3 Utility of seven housekeeping genes was calculated using MLSTest software. **(A)** Comparison of the MLST markers in terms of DP and TE. **(B)** Additive number of DSTs identified by each MLST marker. **(C)** Markers with highest DP in different combinations.

FIGURE S4 Graphical representation of Clonal Complexes (CC) at micro geographical scale.

FIGURE S5 Phylogenetic reconstructions based on the total concatenated sequence using the RAxML method.

FIGURE S6 Phylogenetic trees generated from the alignments of the individual housekeeping genes.

FIGURE S7 Phylogenetic tree based on seven housekeeping genes’ concatenated sequences of 405 isolates from United Kingdom used for micro geographic analyses.

FIGURE S8 Results of the SplitsTree analysis over global population.

FIGURE S9 SplitsTree results at micro geographical scale.

TABLE S1 Analysis of linkage disequilibrium corresponding to recombination events.

TABLE S2 Clonal complex assignment (using the eBURST algorithm) for the total number of reported DSTs.

TABLE S3 eBURST results for the isolates from United Kingdom selected to micro geographic analyses.

TABLE S4 Characterization of possible of recombination events using RDP4 software.
