# Supplementary figures and images for: Dissecting the Heterogeneous Population Genetic Structure of Candida albicans: Limitations and Constraints of the Multilocus Sequence Typing Scheme
Source: Front Microbiol. 2019 May 10;10:1052. doi: 10.3389/fmicb.2019.01052 (PMC6524206; doi:10.3389/fmicb.2019.01052)

# Report of DST by country

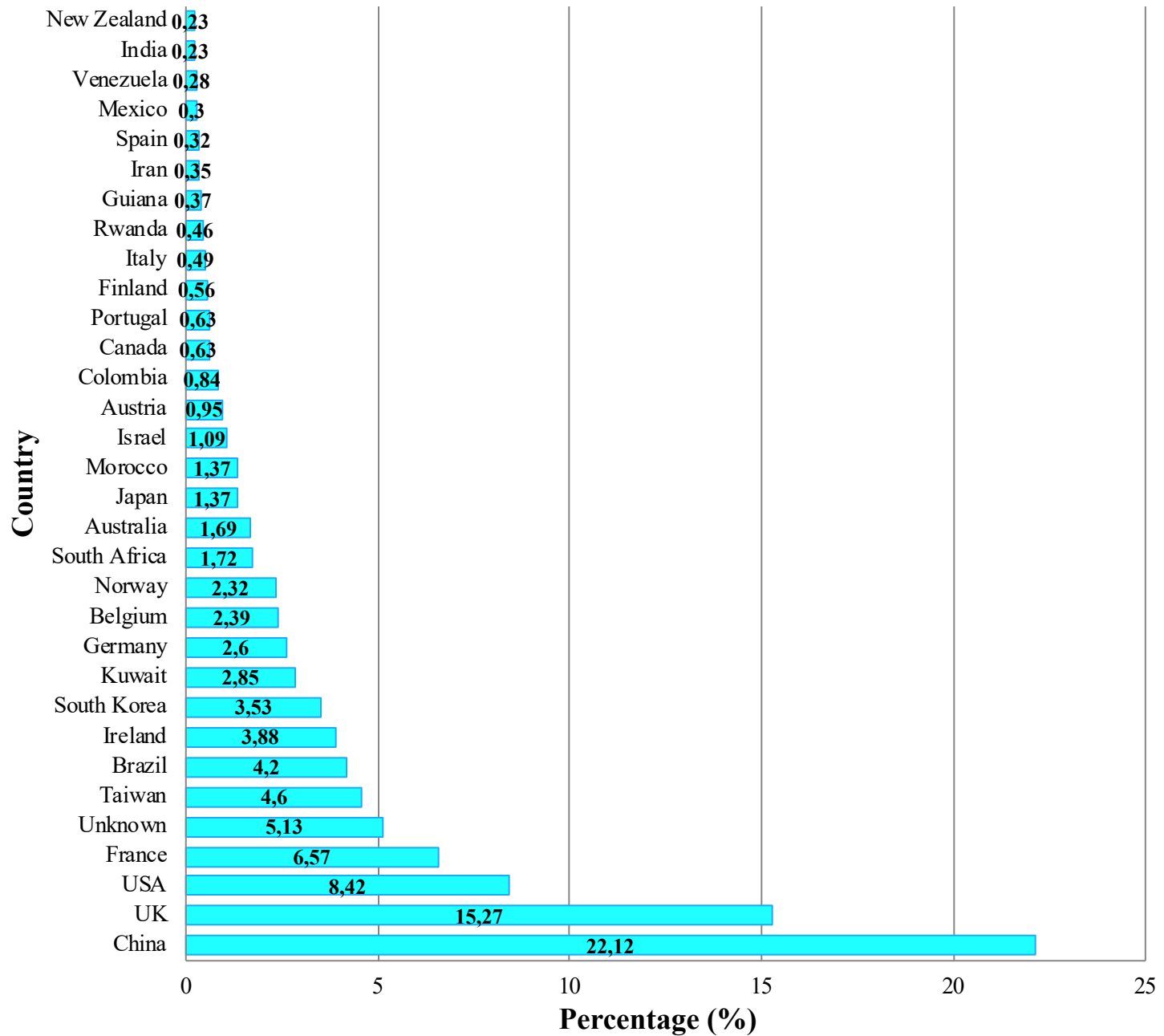

Supplement: FIGURE S1 — Distribution of the reported isolates by country. [file Data_Sheet_1.PDF]

CC-1

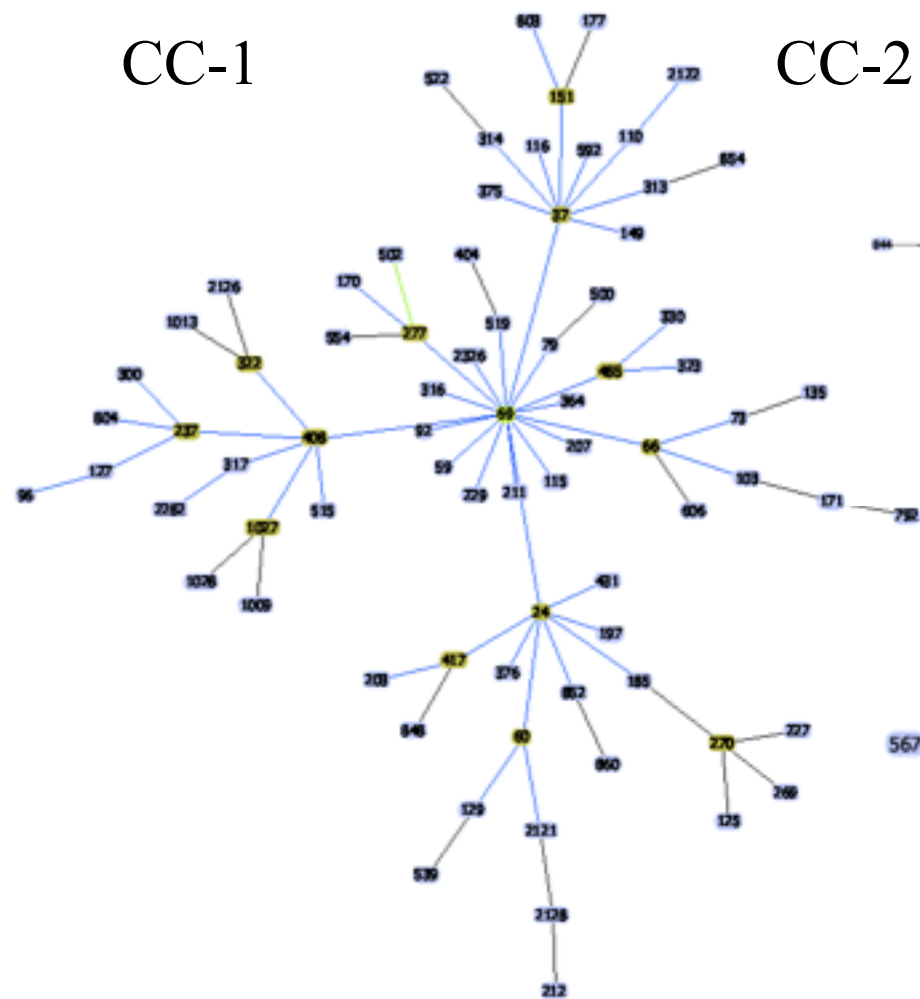

CC-2

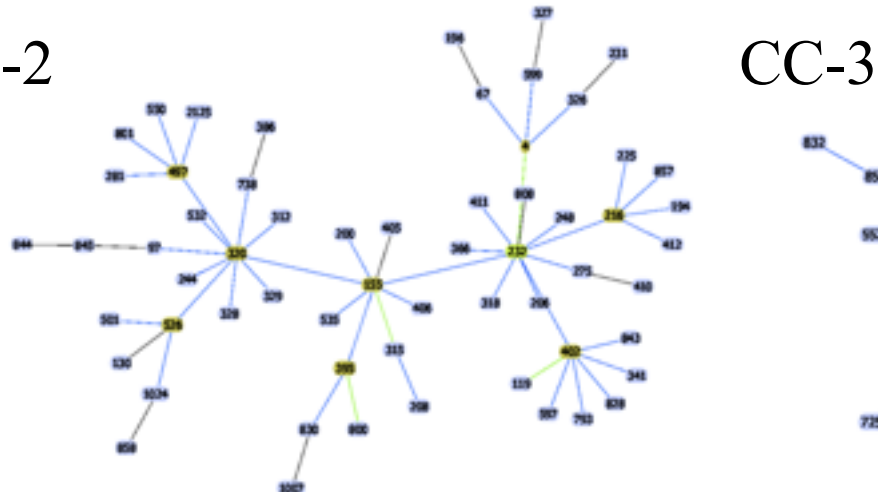

CC-3

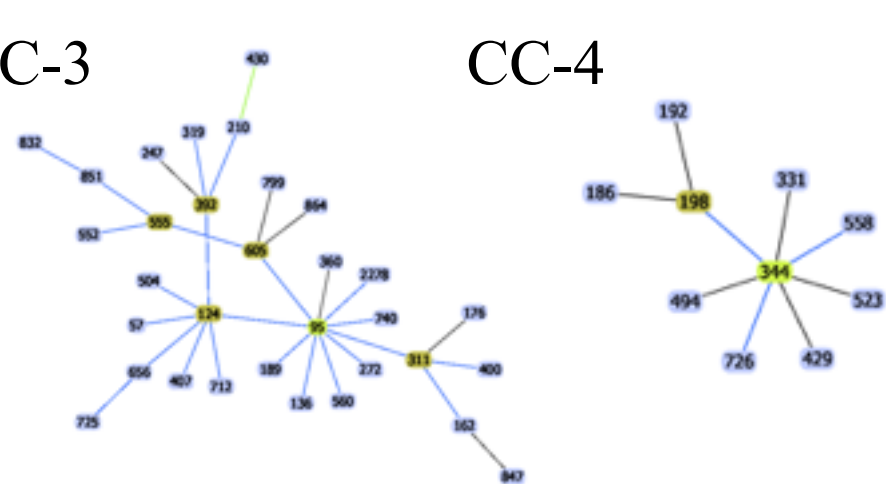

CC-4

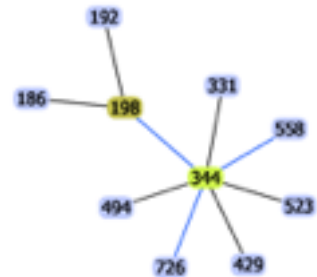

CC-5

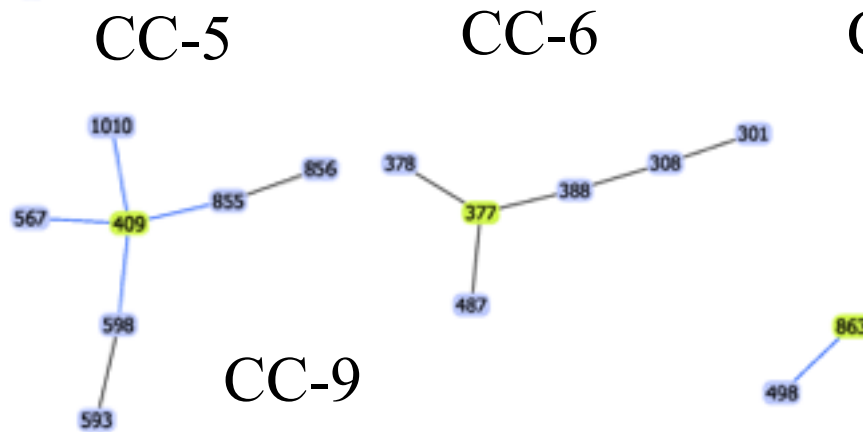

CC-6

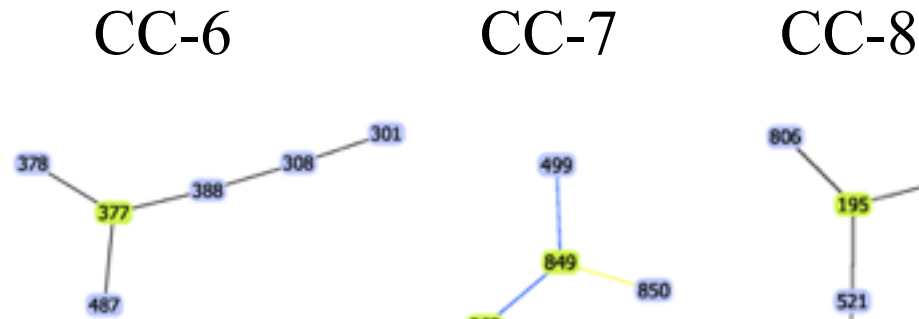

CC-7

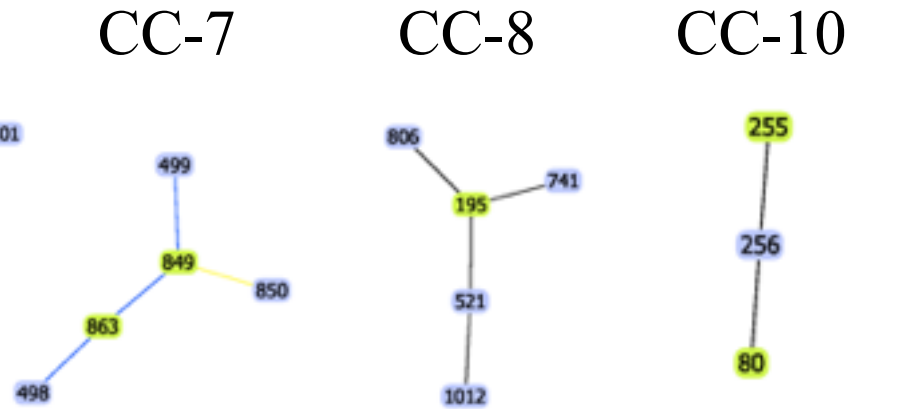

CC-8

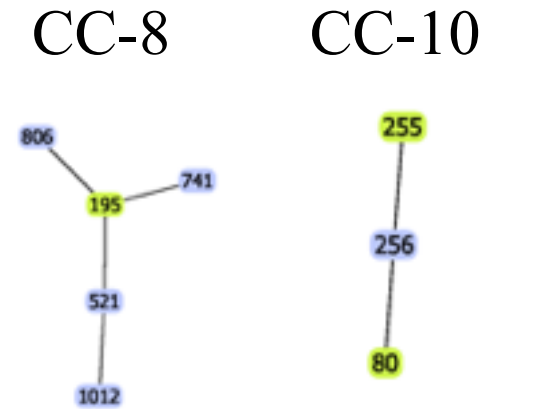

CC-10

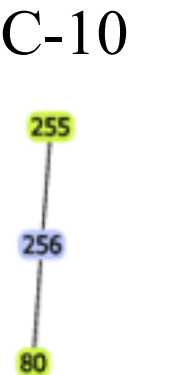

CC-9

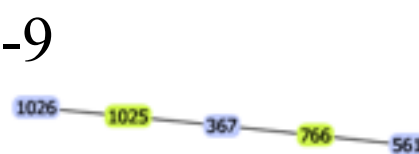

Supplement: FIGURE S4 — Graphical representation of Clonal Complexes (CC) at micro geographical scale. [file Data_Sheet_4.PDF]

Tree scale: 1

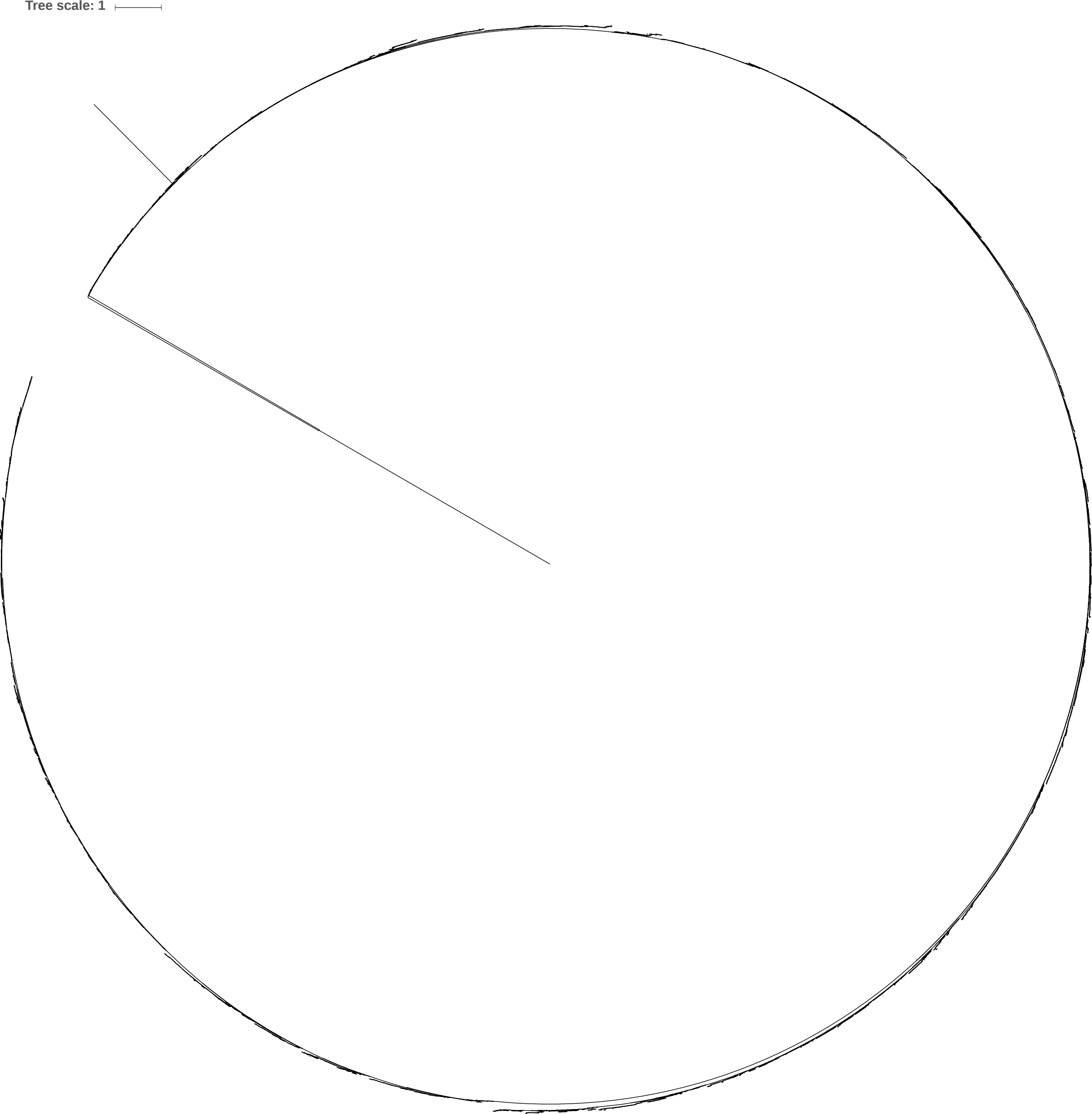

Supplement: FIGURE S5 — Phylogenetic reconstructions based on the total concatenated sequence using the RAxML method. [file Data_Sheet_5.PDF]

# AAT1

Tree scale: 0.01

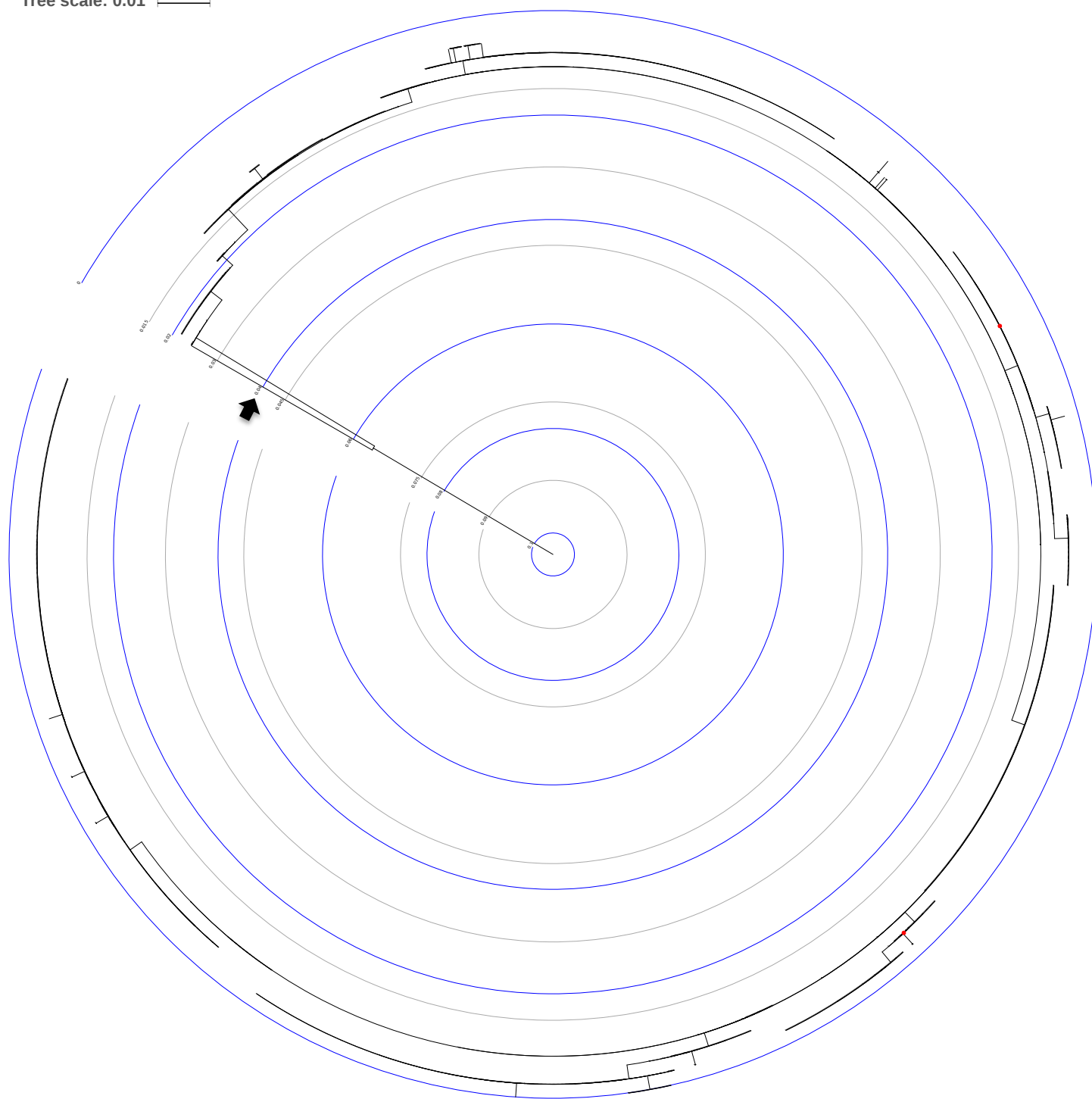

Tree scale: 0.01

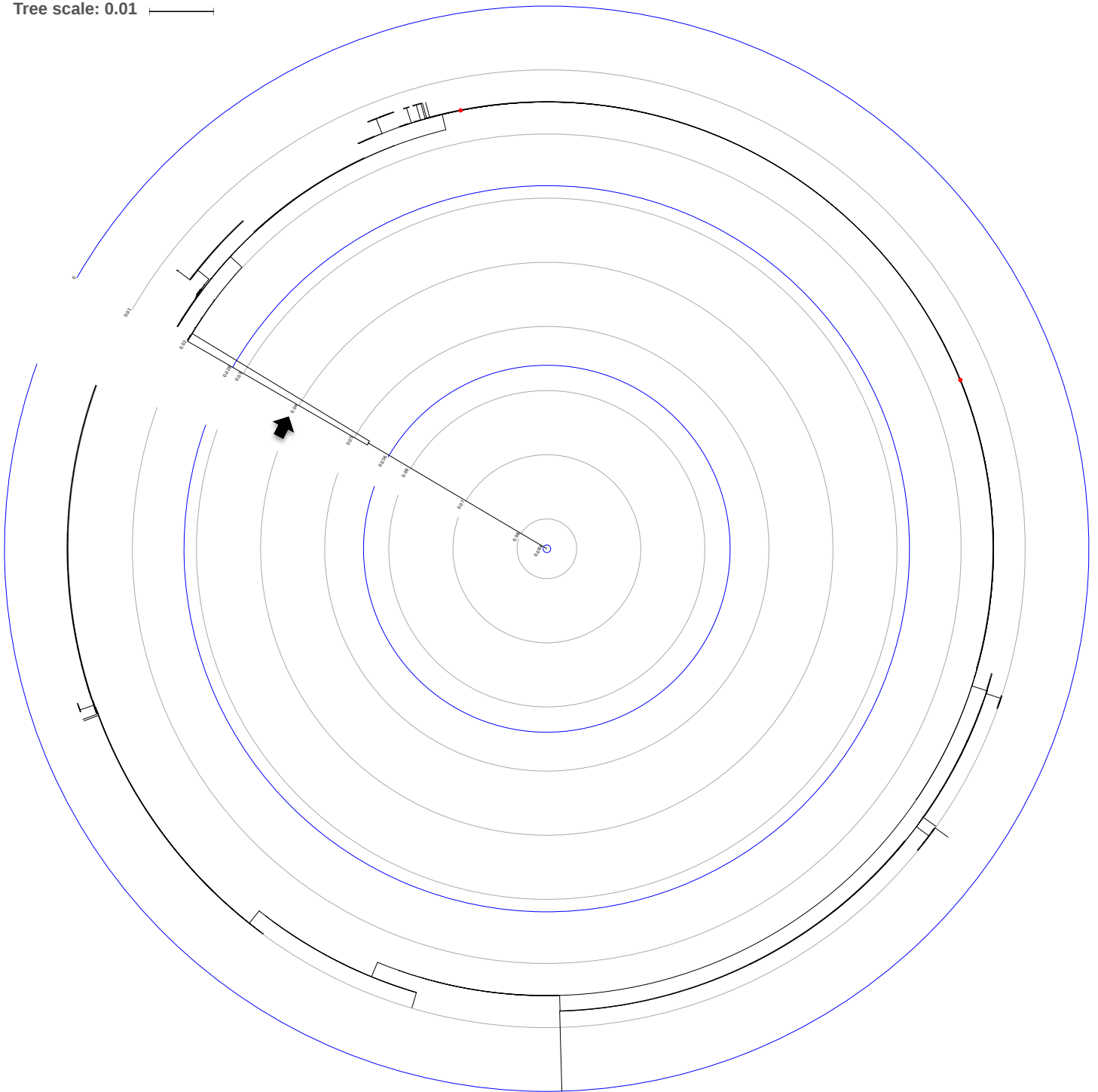

# ADP1

Tree scale: 0.01

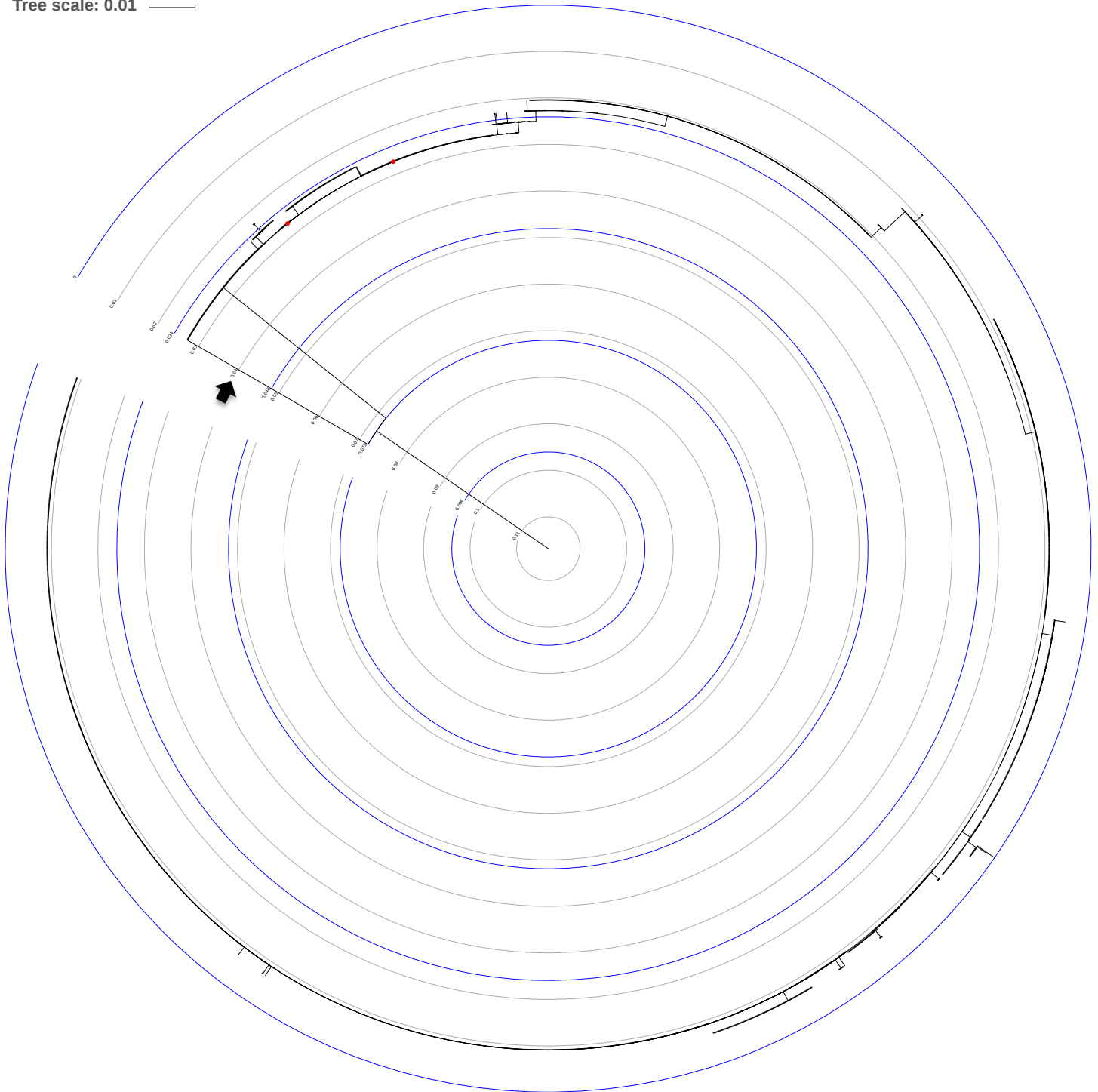

Tree scale: 0.01

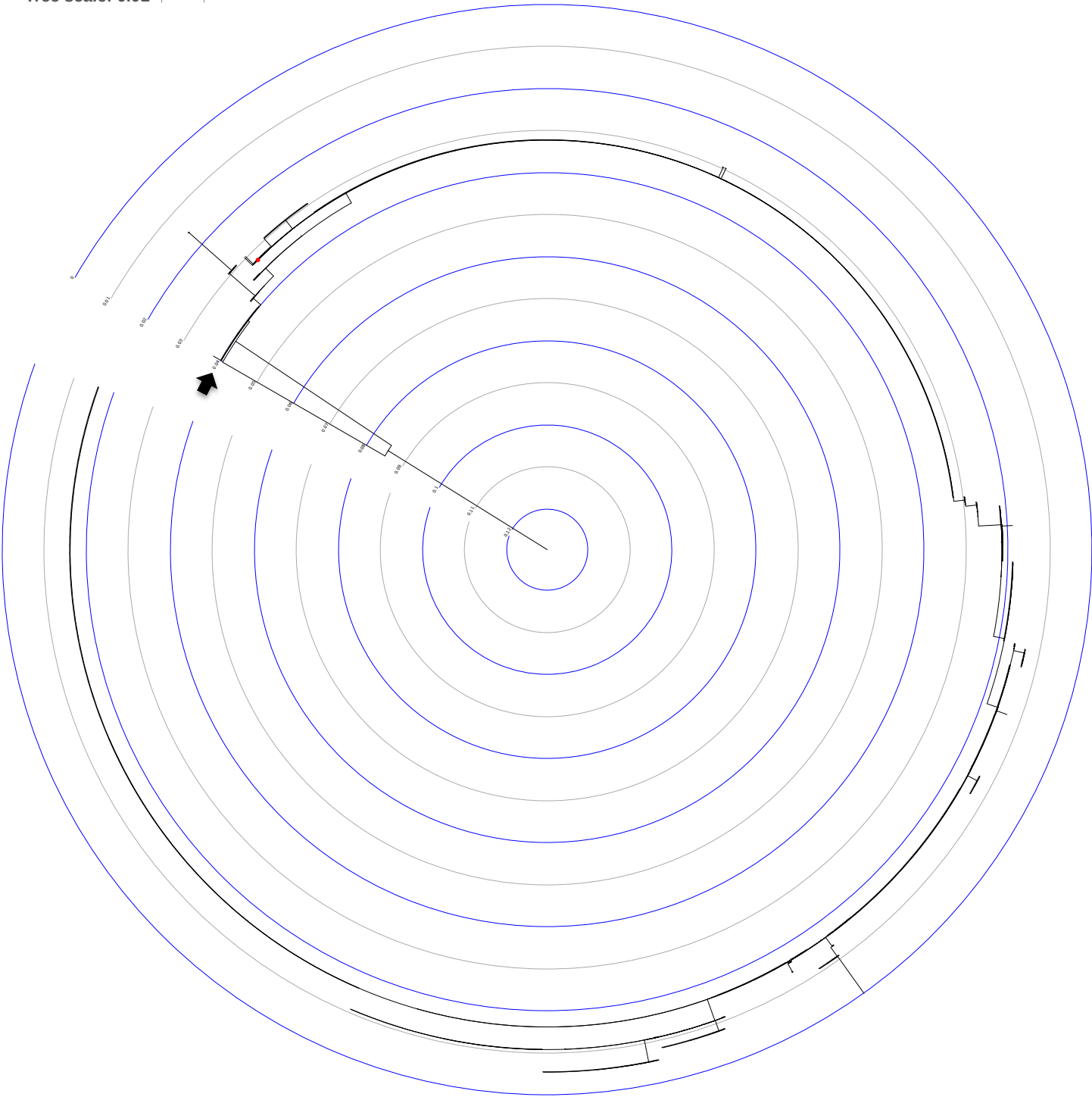

SYA1

Tree scale: 0.01

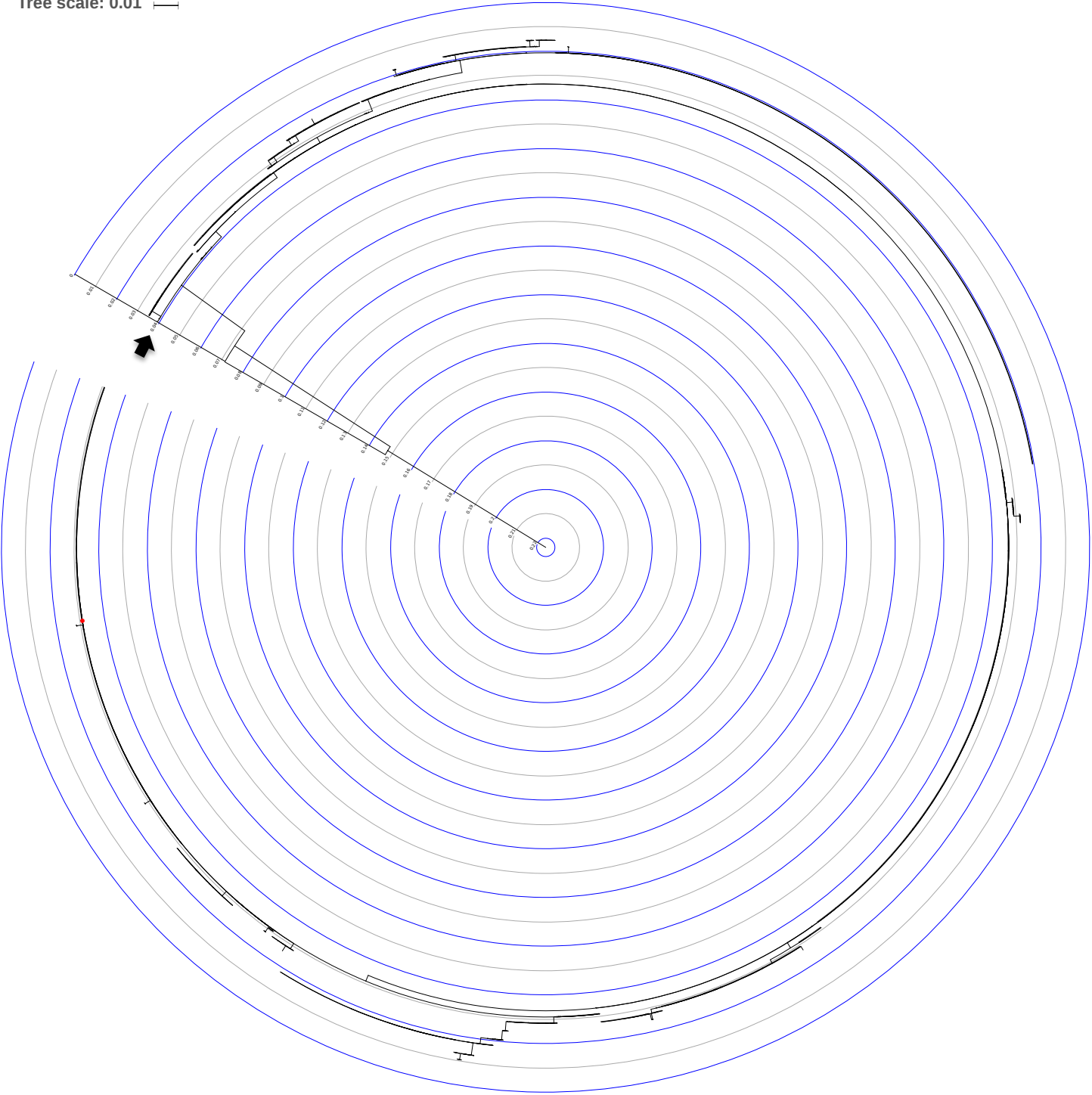

Tree scale: 0.01

VPS13

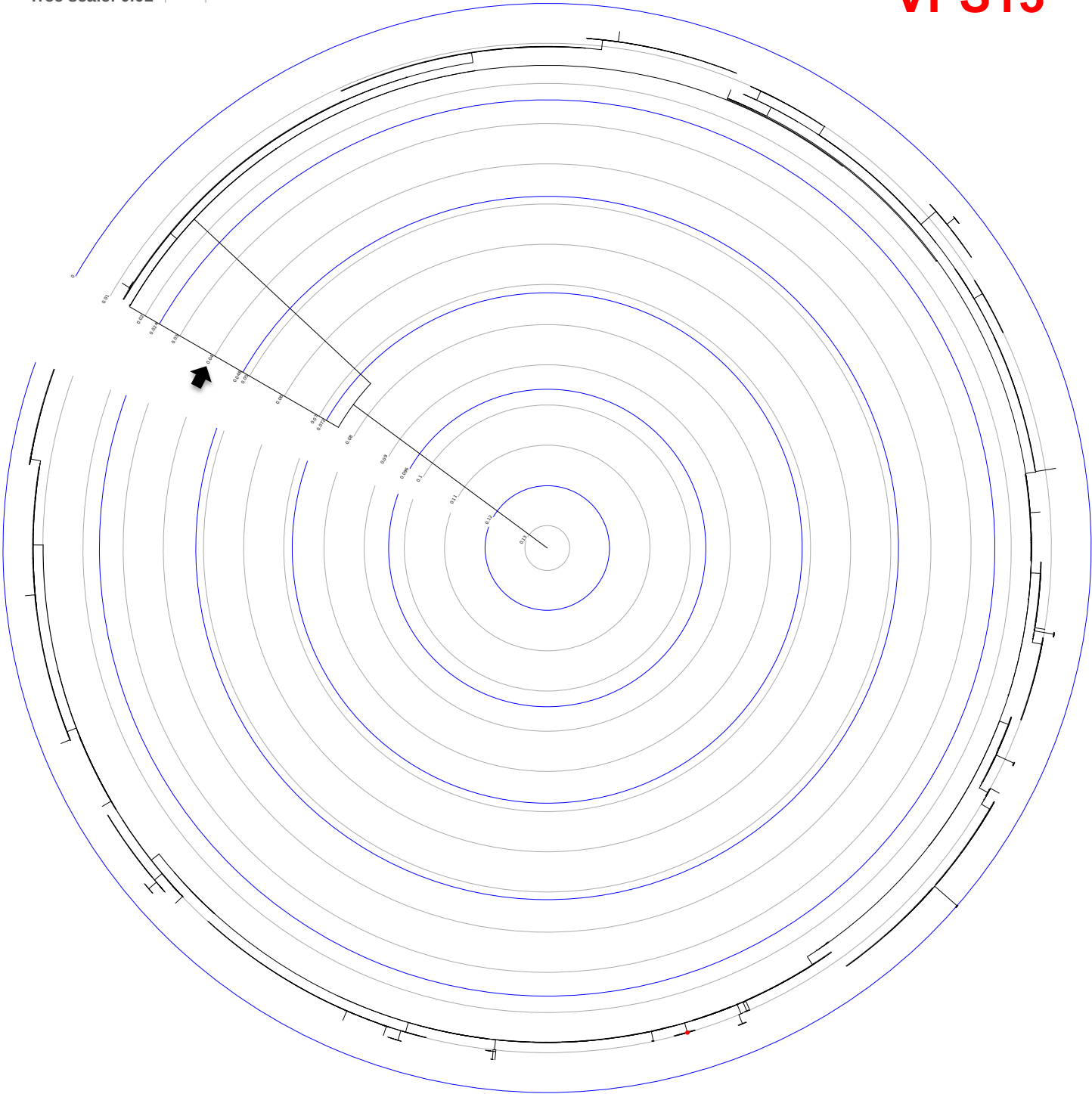

# ZWF1b

Tree scale: 0.01 

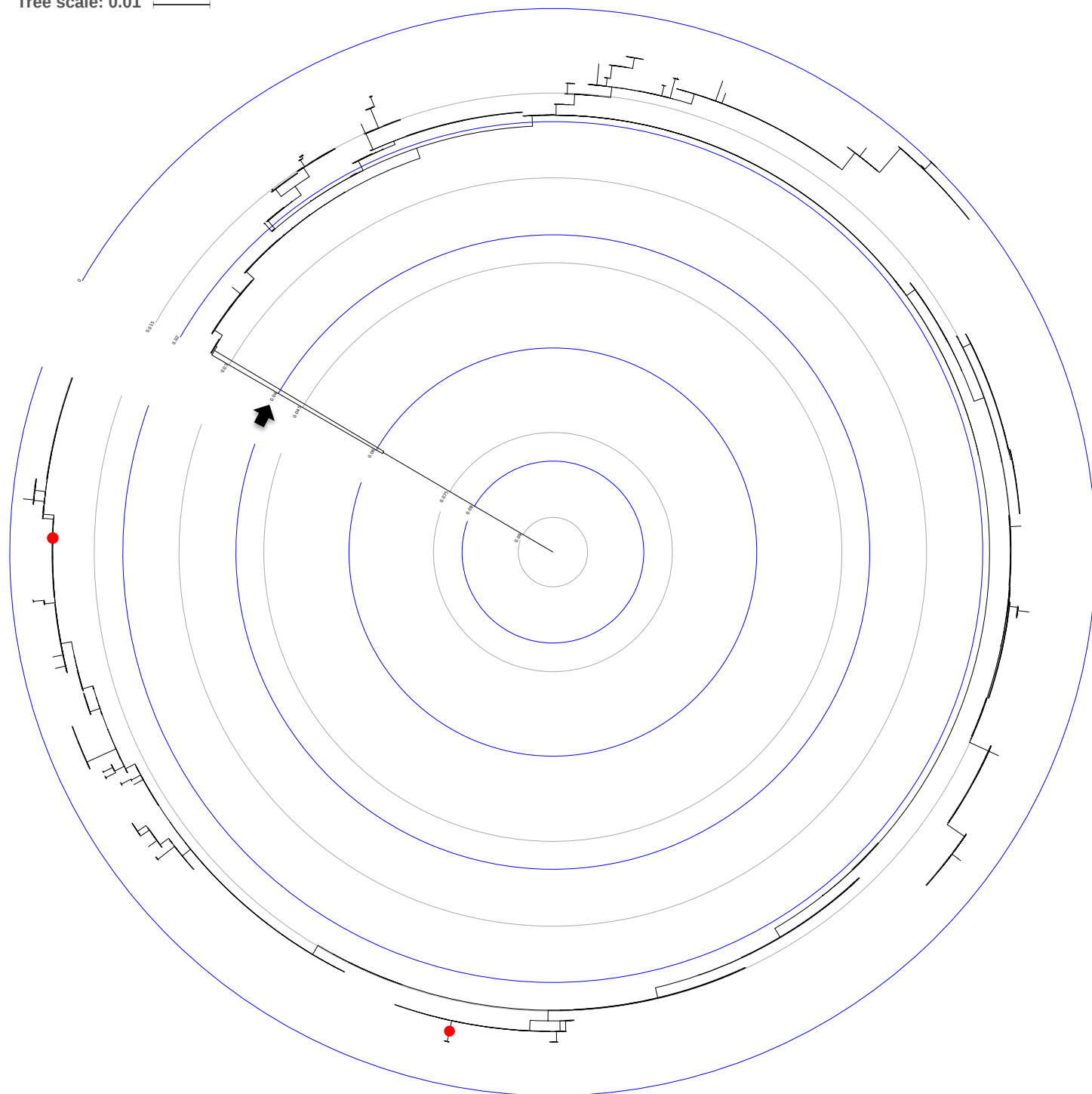

Supplement: FIGURE S6 — Phylogenetic trees generated from the alignments of the individual housekeeping genes. [file Data_Sheet_6.PDF]

Tree scale: 0.001

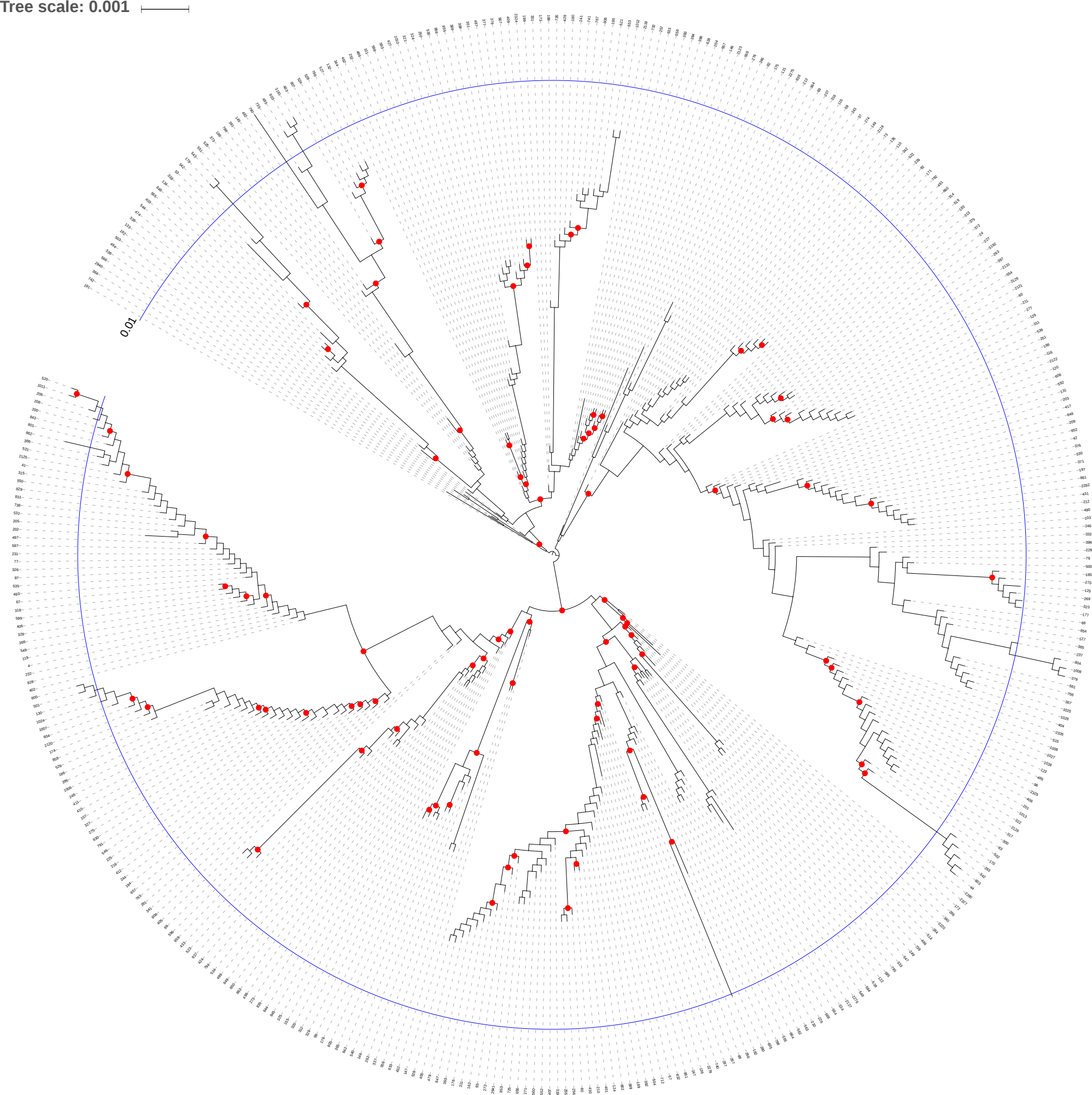

Supplement: FIGURE S7 — Phylogenetic tree based on seven housekeeping genes’ concatenated sequences of 405 isolates from United Kingdom used for micro geographic analyses. [file Data_Sheet_7.PDF]

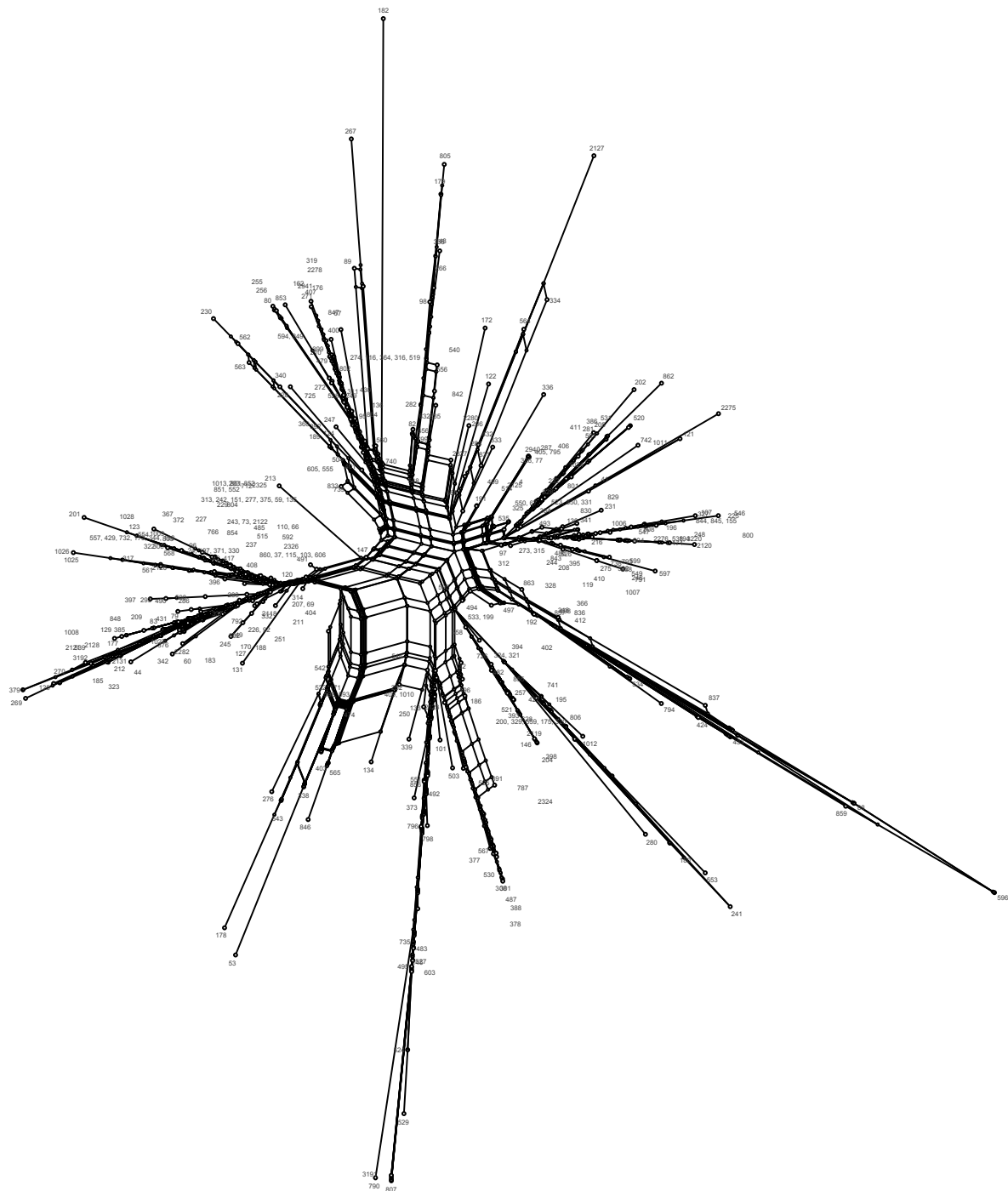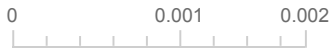

Supplement: FIGURE S9 — SplitsTree results at micro geographical scale. [file Data_Sheet_9.PDF]
