# Supplementary material for: Dissecting the Heterogeneous Population Genetic Structure of Candida albicans: Limitations and Constraints of the Multilocus Sequence Typing Scheme
Source: Front Microbiol. 2019 May 10;10:1052. doi: 10.3389/fmicb.2019.01052 (PMC6524206; doi:10.3389/fmicb.2019.01052)

A.

Total number of DST reported according with year of stablishment

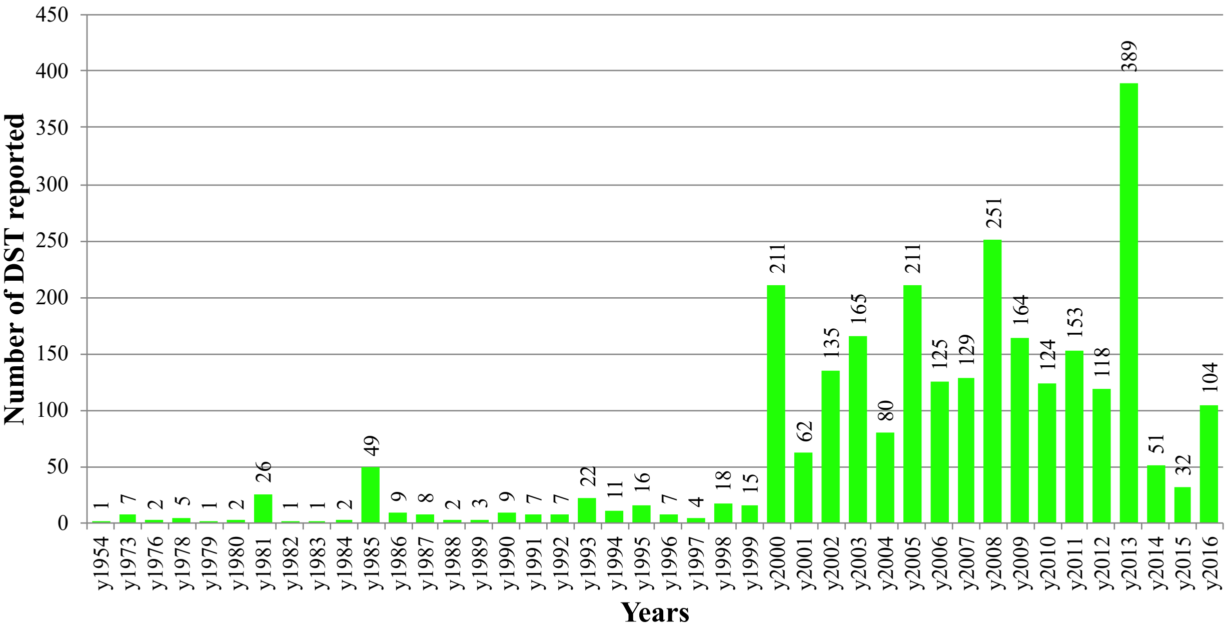

B.

Number of DST reported according with year of stablishment in United Kingdom

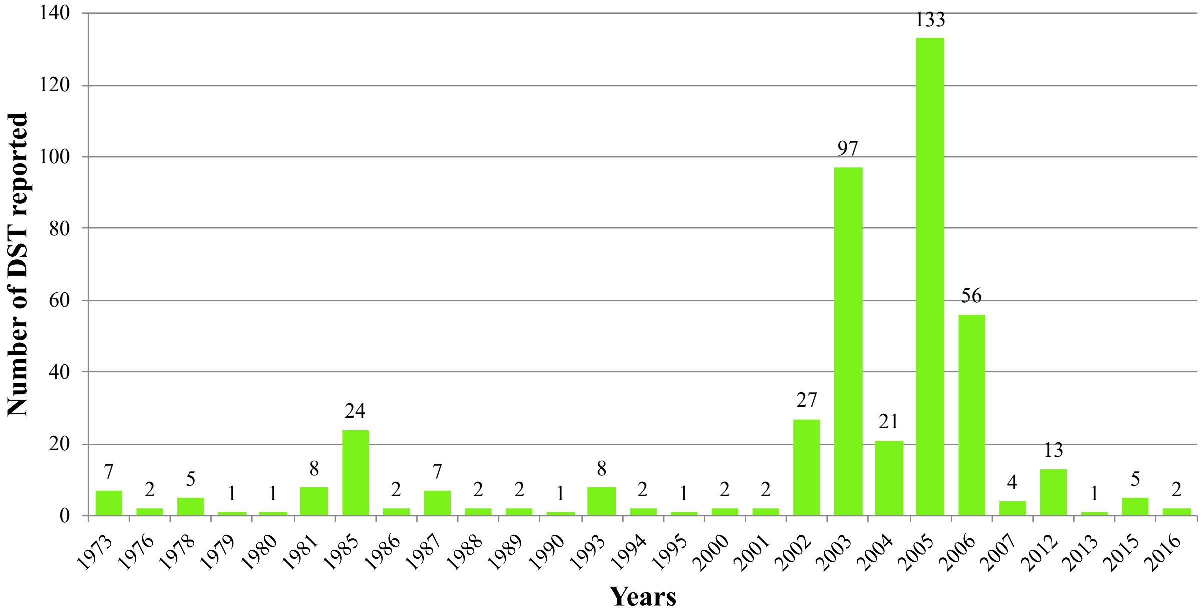

Supplement: FIGURE S2 — The number of isolates reported per year for (A) total of reported isolates; and (B) isolates from United Kingdom. [file Data_Sheet_2.PDF]
