# Supplementary material for: Dissecting the Heterogeneous Population Genetic Structure of Candida albicans: Limitations and Constraints of the Multilocus Sequence Typing Scheme
Source: Front Microbiol. 2019 May 10;10:1052. doi: 10.3389/fmicb.2019.01052 (PMC6524206; doi:10.3389/fmicb.2019.01052)
Supplement: FIGURE S3 — Utility of seven housekeeping genes was calculated using MLSTest software. (A) Comparison of the MLST markers in terms of DP and TE. (B) Additive number of DSTs identified by each MLST marker. (C) Markers with highest DP in different combinations. [file Data_Sheet_3.PDF]

A.

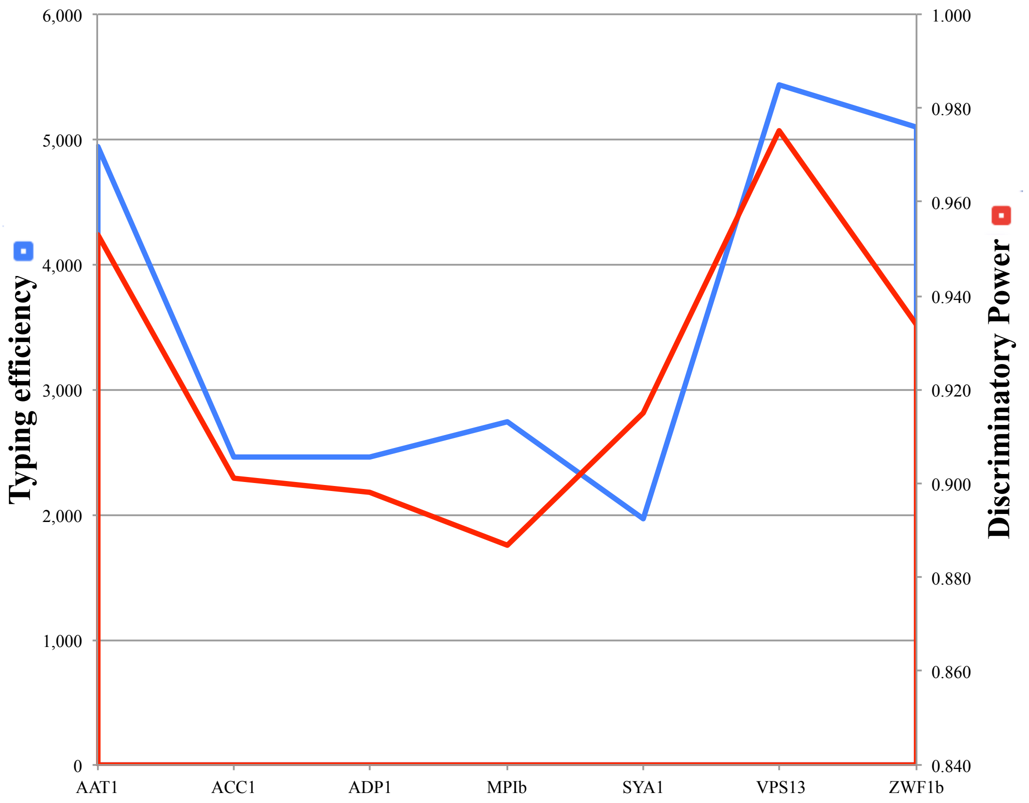

B.

Number of loci (number of combinations)	Minimum number of STs found	Mean number of STs found	Maximum number of STs found
1 (7)	113	2,002,857	299
2 (21)	532	8,171,429	1145
3 (35)	1000	1506	1858
4 (35)	1000	2,083,314	2394
5 (21)	1000	2,591,572	2849
6 (7)	1000	3,058,572	3191
7 (1)	1000	3482	3482

C.

Number of loci	Maximum number of ST	Combinations analyzed	Housekeeping genes
2	1145	21	VPS13, ZWF1b
3	1858	35	AAT1a, VPS13, ZWF1b
4	2394	35	AAT1a, ACC1, VPS13, ZWF1b
5	2849	21	AAT1a, ACC1, SYA1, VPS13, ZWF1b
6	3191	7	AAT1a, ACC1, ADP1, SYA1, VPS13, ZWF1b
7	3482	1	AAT1a, ACC1, ADP1, MPIb, SYA1, VPS13, ZWF1b
