# Supplementary material for: Dissecting the Heterogeneous Population Genetic Structure of Candida albicans: Limitations and Constraints of the Multilocus Sequence Typing Scheme
Source: Front Microbiol. 2019 May 10;10:1052. doi: 10.3389/fmicb.2019.01052 (PMC6524206; doi:10.3389/fmicb.2019.01052)
Supplement: FIGURE S8 — Results of the SplitsTree analysis over global population. [file Data_Sheet_8.PDF]

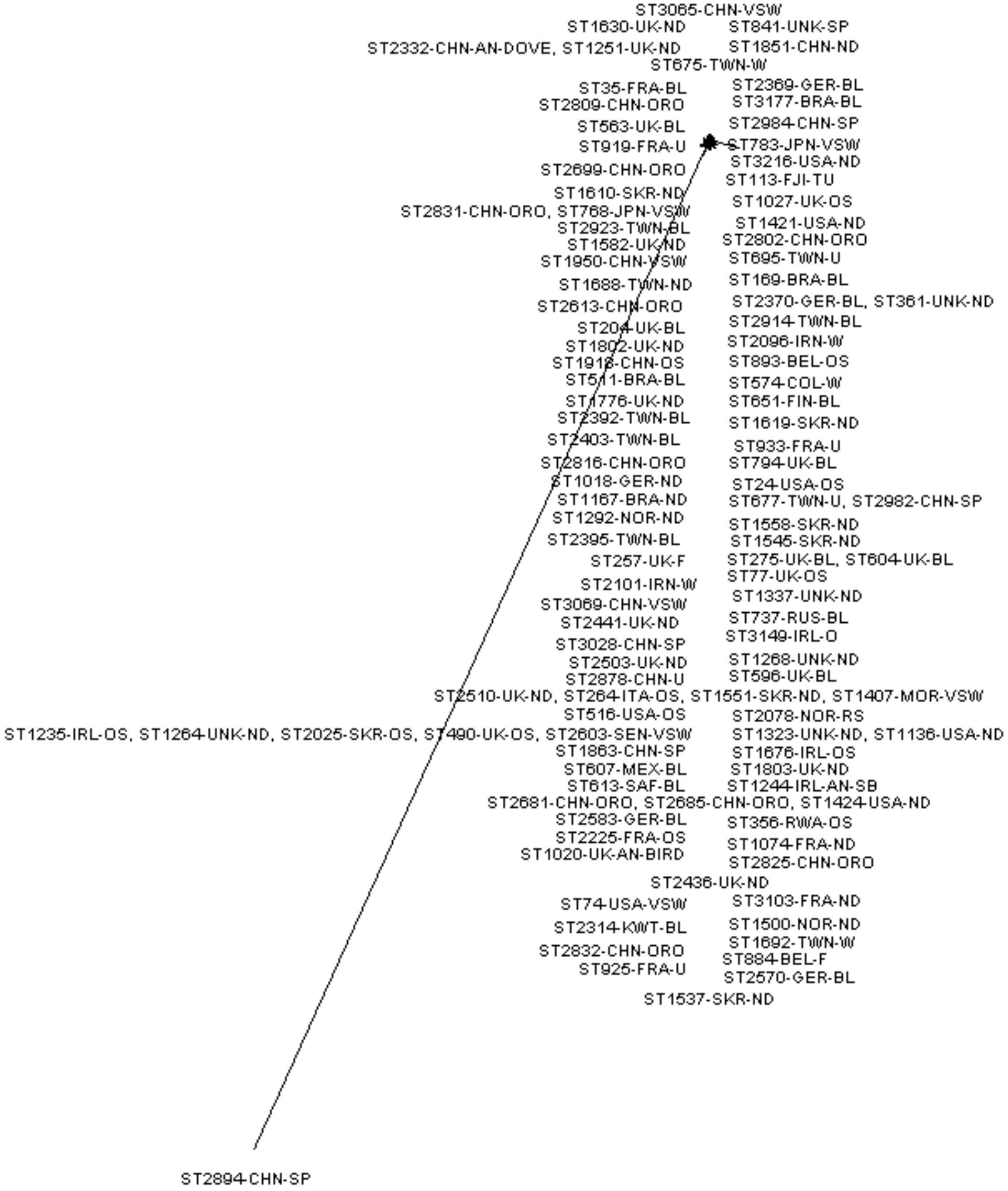

ST3065-CHN-VSW
ST1630-UK-ND
ST841-UNK-SP
ST2332-CHN-AN-DOVE, ST1251-UK-ND
ST1851-CHN-ND
ST675-TWN-W
ST35-FRA-BL
ST2369-GER-BL
ST2809-CHN-ORO
ST3177-BRA-BL
ST563-UK-BL
ST2984-CHN-SP
ST919-FRA-U
ST783-JPN-VSW
ST2699-CHN-ORO
ST3216-USA-ND
ST113-FJI-TU
ST1610-SKR-ND
ST1027-UK-OS
ST2831-CHN-ORO, ST768-JPN-VSW
ST1421-USA-ND
ST2923-TWN-BL
ST2802-CHN-ORO
ST1582-UK-ND
ST1950-CHN-VSW
ST695-TWN-U
ST1688-TWN-ND
ST169-BRA-BL
ST2613-CHN-ORO
ST2370-GER-BL, ST361-UNK-ND
ST204-UK-BL
ST2914-TWN-BL
ST1802-UK-ND
ST2096-IRN-W
ST1918-CHN-OS
ST893-BEL-OS
ST511-BRA-BL
ST574-COL-W
ST1776-UK-ND
ST651-FIN-BL
ST2392-TWN-BL
ST1619-SKR-ND
ST2403-TWN-BL
ST933-FRA-U
ST2816-CHN-ORO
ST794-UK-BL
ST1018-GER-ND
ST24-USA-OS
ST1167-BRA-ND
ST677-TWN-U, ST2982-CHN-SP
ST1292-NOR-ND
ST1558-SKR-ND
ST2395-TWN-BL
ST1545-SKR-ND
ST257-UK-F
ST275-UK-BL, ST604-UK-BL
ST2101-IRN-W
ST77-UK-OS
ST3069-CHN-VSW
ST1337-UNK-ND
ST2441-UK-ND
ST737-RUS-BL
ST3149-IRL-O
ST3028-CHN-SP
ST1268-UNK-ND
ST2503-UK-ND
ST2878-CHN-U
ST596-UK-BL
ST2510-UK-ND, ST264-ITA-OS, ST1551-SKR-ND, ST1407-MOR-VSW
ST516-USA-OS
ST2078-NOR-RS
ST1235-IRL-OS, ST1264-UNK-ND, ST2025-SKR-OS, ST490-UK-OS, ST2603-SEN-VSW
ST1323-UNK-ND, ST1136-USA-ND
ST1863-CHN-SP
ST1676-IRL-OS
ST607-MEX-BL
ST1803-UK-ND
ST613-SAF-BL
ST1244-IRL-AN-SB
ST2681-CHN-ORO, ST2685-CHN-ORO, ST1424-USA-ND
ST2583-GER-BL
ST356-RWA-OS
ST2225-FRA-OS
ST1074-FRA-ND
ST1020-UK-AN-BIRD
ST2825-CHN-ORO
ST2436-UK-ND
ST74-USA-VSW
ST3103-FRA-ND
ST2314-KWT-BL
ST1500-NOR-ND
ST2832-CHN-ORO
ST1692-TWN-W
ST925-FRA-U
ST884-BEL-F
ST2570-GER-BL
ST1537-SKR-ND
ST2894-CHN-SP

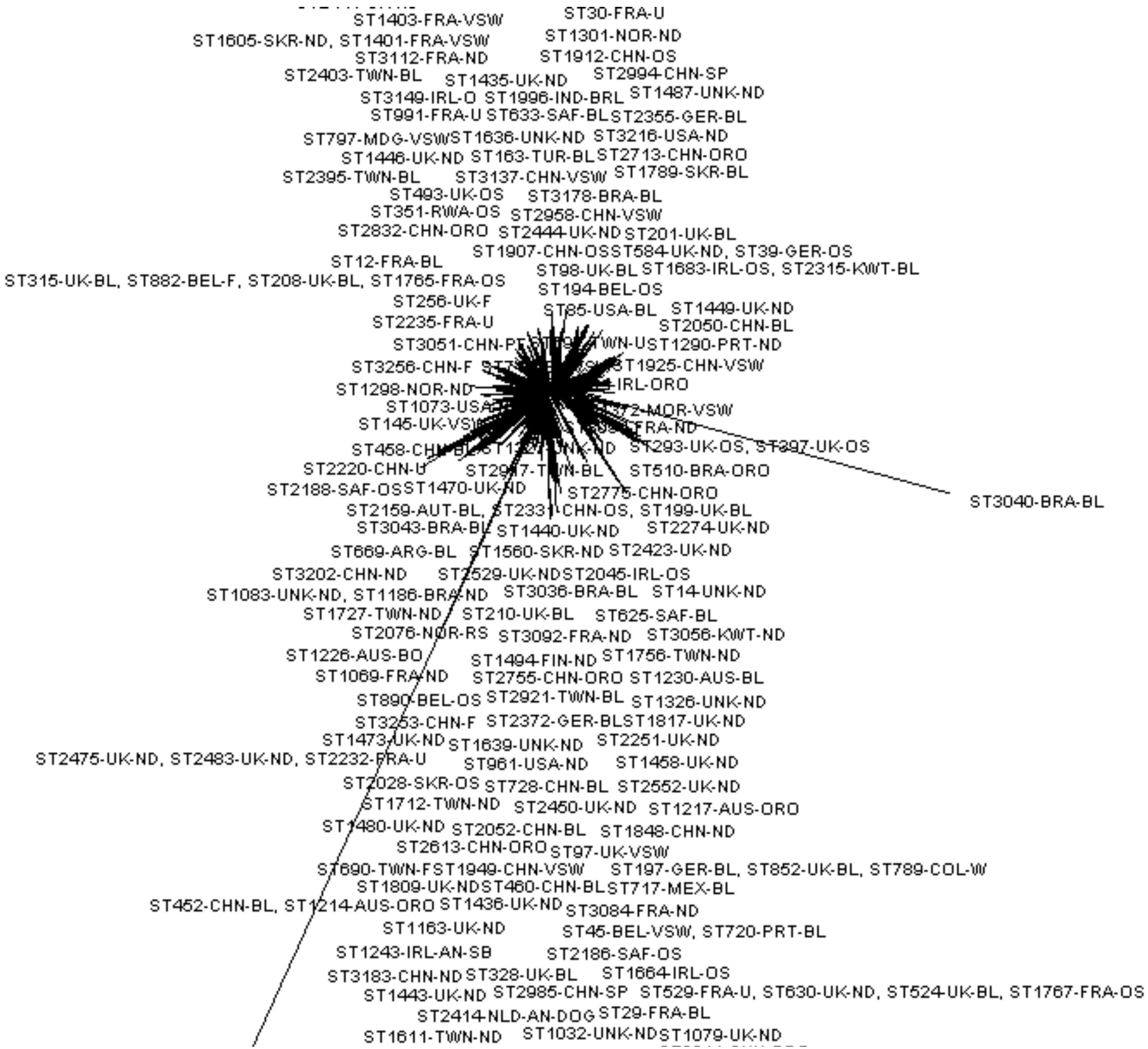

ST1403-FRA-VSW
ST30-FRA-U
ST1605-SKR-ND, ST1401-FRA-VSW
ST1301-NOR-ND
ST3112-FRA-ND
ST1912-CHN-OS
ST2403-TWN-BL
ST1435-UK-ND
ST2994-CHN-SP
ST3149-IRL-O
ST1996-IND-BRL
ST1487-UNK-ND
ST991-FRA-U
ST633-SAF-BL
ST2355-GER-BL
ST797-MDG-VSW
ST1636-UNK-ND
ST3216-USA-ND
ST1446-UK-ND
ST163-TUR-BL
ST2713-CHN-ORO
ST2395-TWN-BL
ST3137-CHN-VSW
ST1789-SKR-BL
ST493-UK-OS
ST3178-BRA-BL
ST351-RWA-OS
ST2958-CHN-VSW
ST2832-CHN-ORO
ST2444-UK-ND
ST201-UK-BL
ST1907-CHN-OS
ST584-UK-ND, ST39-GER-OS
ST12-FRA-BL
ST98-UK-BL
ST1683-IRL-OS, ST2315-KWT-BL
ST315-UK-BL, ST882-BEL-F, ST208-UK-BL, ST1765-FRA-OS
ST194-BEL-OS
ST256-UK-F
ST1449-UK-ND
ST2235-FRA-U
ST2050-CHN-BL
ST1290-PRT-ND
ST3256-CHN-F
ST1925-CHN-VSW
ST1298-NOR-ND
ST293-UK-OS, ST397-UK-OS
ST510-BRA-ORO
ST2220-CHN-U
ST3040-BRA-BL
ST2188-SAF-OS
ST1470-UK-ND
ST2775-CHN-ORO
ST2159-AUT-BL, ST2331-CHN-OS, ST199-UK-BL
ST3043-BRA-BL
ST1440-UK-ND
ST2274-UK-ND
ST669-ARG-BL
ST1560-SKR-ND
ST2423-UK-ND
ST3202-CHN-ND
ST2529-UK-ND
ST2045-IRL-OS
ST1083-UNK-ND, ST1186-BRA-ND
ST3036-BRA-BL
ST14-UNK-ND
ST1727-TWN-ND
ST210-UK-BL
ST625-SAF-BL
ST2076-NOR-RS
ST3092-FRA-ND
ST3056-KWT-ND
ST1226-AUS-BO
ST1494-FIN-ND
ST1756-TWN-ND
ST1069-FRA-ND
ST2755-CHN-ORO
ST1230-AUS-BL
ST890-BEL-OS
ST2921-TWN-BL
ST1326-UNK-ND
ST3253-CHN-F
ST2372-GER-BL
ST1817-UK-ND
ST1473-UK-ND
ST1639-UNK-ND
ST2251-UK-ND
ST2475-UK-ND, ST2483-UK-ND, ST2232-FRA-U
ST961-USA-ND
ST1458-UK-ND
ST2028-SKR-OS
ST728-CHN-BL
ST2552-UK-ND
ST1712-TWN-ND
ST2450-UK-ND
ST1217-AUS-ORO
ST1480-UK-ND
ST2052-CHN-BL
ST1848-CHN-ND
ST2613-CHN-ORO
ST97-UK-VSW
ST690-TWN-F
ST1949-CHN-VSW
ST197-GER-BL, ST852-UK-BL, ST789-COL-W
ST1809-UK-ND
ST460-CHN-BL
ST717-MEX-BL
ST452-CHN-BL, ST1214-AUS-ORO
ST1436-UK-ND
ST3084-FRA-ND
ST1163-UK-ND
ST45-BEL-VSW, ST720-PRT-BL
ST1243-IRL-AN-SB
ST2186-SAF-OS
ST3183-CHN-ND
ST328-UK-BL
ST1664-IRL-OS
ST1443-UK-ND
ST2985-CHN-SP
ST529-FRA-U, ST630-UK-ND, ST524-UK-BL, ST1767-FRA-OS
ST2414-NLD-AN-DOG
ST29-FRA-BL
ST1611-TWN-ND
ST1032-UNK-ND
ST1079-UK-ND

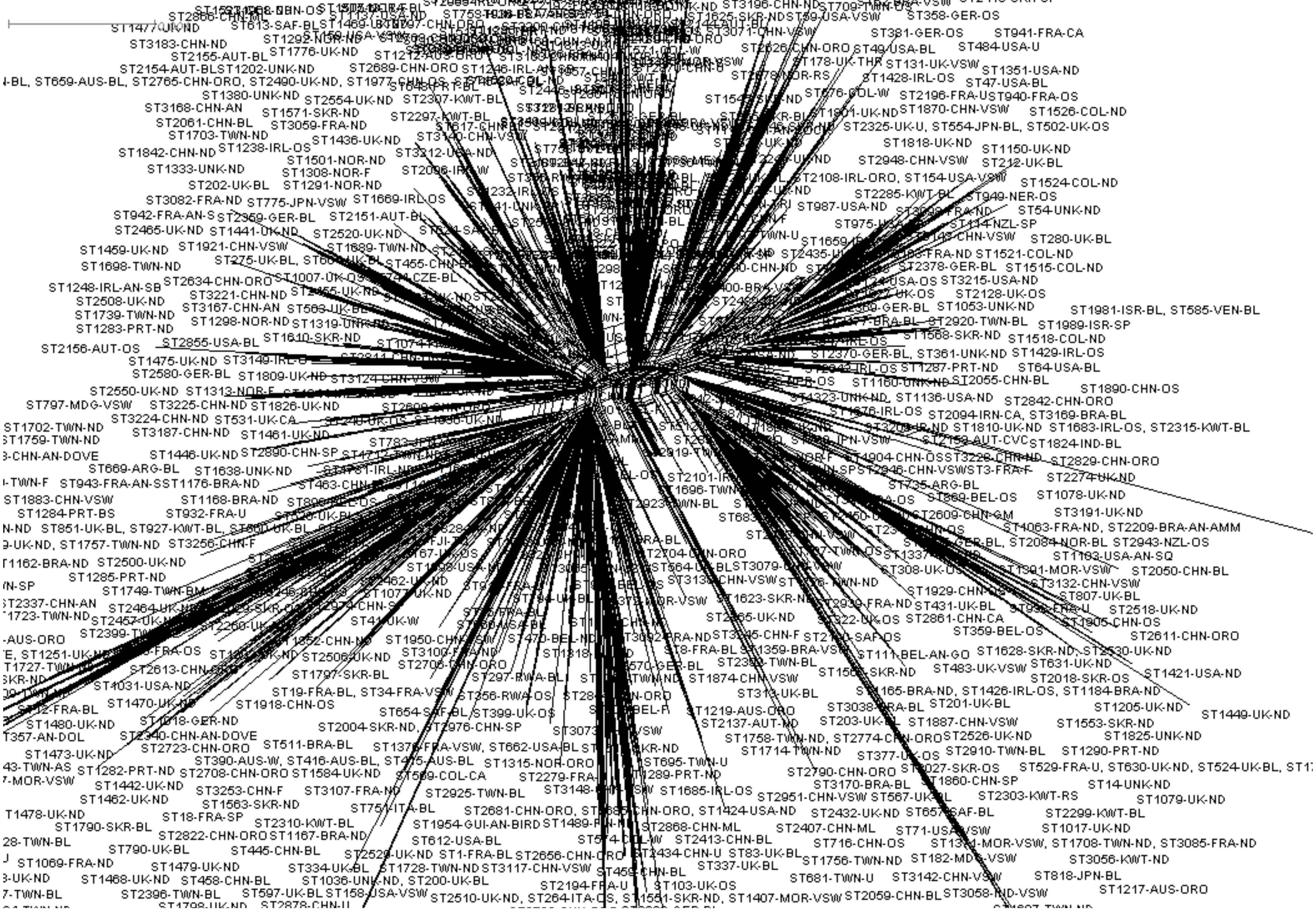

ST1477-UK-ND
ST3183-CHN-ND
ST2155-AUT-BL
ST2154-AUT-BL
ST1202-UNK-ND
ST1380-UNK-ND
ST3168-CHN-AN
ST2061-CHN-BL
ST1703-TWN-ND
ST1842-CHN-ND
ST1333-UNK-ND
ST202-UK-BL
ST3082-FRA-ND
ST942-FRA-AN-S
ST2465-UK-ND
ST1459-UK-ND
ST1698-TWN-ND
ST1248-IRL-AN-SB
ST2508-UK-ND
ST1739-TWN-ND
ST1283-PRT-ND
ST2156-AUT-OS
ST1475-UK-ND
ST2580-GER-BL
ST2550-UK-ND
ST797-MDG-VSW
ST3225-CHN-ND
ST3224-CHN-ND
ST3187-CHN-ND
ST1446-UK-ND
ST669-ARG-BL
ST943-FRA-AN-S
ST1176-BRA-ND
ST1883-CHN-VSW
ST1284-PRT-BS
ST851-UK-BL, ST927-KWT-BL
ST3256-CHN-F
ST2500-UK-ND
ST1285-PRT-ND
ST1749-TWN-BM
ST1031-USA-ND
ST1470-UK-ND
ST1480-UK-ND
ST1473-UK-ND
ST1282-PRT-ND
ST1442-UK-ND
ST1462-UK-ND
ST1790-SKR-BL
ST1069-FRA-ND
ST1468-UK-ND
ST2396-TWN-BL
ST1798-UK-ND
ST2878-CHN-U
ST1613-SAF-BL
ST1292-NOR-ND
ST1776-UK-ND
ST2689-CHN-ORO
ST2554-UK-ND
ST2307-KWT-BL
ST1571-SKR-ND
ST3059-FRA-ND
ST2297-KWT-BL
ST1436-UK-ND
ST3140-CHN-VSW
ST1238-IRL-OS
ST1501-NOR-ND
ST3212-USA-ND
ST1308-NOR-F
ST2096-IRL-W
ST1291-NOR-ND
ST775-JPN-VSW
ST1669-IRL-OS
ST2369-GER-BL
ST2151-AUT-BL
ST1441-UK-ND
ST2520-UK-ND
ST1921-CHN-VSW
ST1689-TWN-ND
ST2634-CHN-ORO
ST3221-CHN-ND
ST3167-CHN-AN
ST1298-NOR-ND
ST1319-UNK-ND
ST2855-USA-BL
ST1610-SKR-ND
ST3149-IRL-U
ST1809-UK-ND
ST3124-CHN-VSW
ST1313-NOR-F
ST1826-UK-ND
ST531-UK-CA
ST1461-UK-ND
ST2890-CHN-SP
ST1638-UNK-ND
ST1168-BRA-ND
ST932-FRA-U
ST2500-UK-ND
ST1077-UK-ND
ST41-UK-W
ST1950-CHN-VSW
ST3100-FRA-ND
ST2705-CHN-ORO
ST2506-UK-ND
ST1797-SKR-BL
ST19-FRA-BL, ST34-FRA-VSW
ST1918-CHN-OS
ST654-SAF-BL
ST399-UK-OS
ST1018-GER-ND
ST2340-CHN-AN-DOVE
ST2723-CHN-ORO
ST511-BRA-BL
ST2004-SKR-ND, ST2976-CHN-SP
ST1376-FRA-VSW, ST662-USA-BL
ST390-AUS-W, ST416-AUS-BL, ST425-AUS-BL
ST2708-CHN-ORO
ST1584-UK-ND
ST569-COL-CA
ST3253-CHN-F
ST3107-FRA-ND
ST2925-TWN-BL
ST1563-SKR-ND
ST751-ITA-BL
ST18-FRA-SP
ST2310-KWT-BL
ST1954-GUI-AN-BIRD
ST2822-CHN-ORO
ST1167-BRA-ND
ST612-USA-BL
ST790-UK-BL
ST445-CHN-BL
ST2529-UK-ND
ST1-FRA-BL
ST2656-CHN-ORO
ST1479-UK-ND
ST334-UK-BL
ST1728-TWN-ND
ST3117-CHN-VSW
ST458-CHN-BL
ST1036-UNK-ND, ST200-UK-BL
ST597-UK-BL
ST158-USA-VSW
ST2510-UK-ND, ST264-ITA-OS, ST1551-SKR-ND, ST1407-MOR-VSW
ST358-GER-OS
ST381-GER-OS
ST941-FRA-CA
ST2626-CHN-ORO
ST49-USA-BL
ST484-USA-U
ST178-UK-THR
ST131-UK-VSW
ST1351-USA-ND
ST1428-IRL-OS
ST47-USA-BL
ST2196-FRA-U
ST940-FRA-OS
ST1870-CHN-VSW
ST1526-COL-ND
ST2325-UK-U, ST554-JPN-BL, ST502-UK-OS
ST1818-UK-ND
ST1150-UK-ND
ST2948-CHN-VSW
ST212-UK-BL
ST2108-IRL-ORO, ST154-USA-VSW
ST1524-COL-ND
ST2285-KWT-BL
ST949-NER-OS
ST987-USA-ND
ST54-UNK-ND
ST114-NZL-SP
ST280-UK-BL
ST1521-COL-ND
ST2378-GER-BL
ST1515-COL-ND
ST3215-USA-ND
ST2128-UK-OS
ST1053-UNK-ND
ST1981-ISR-BL, ST585-VEN-BL
ST2920-TWN-BL
ST1989-ISR-SP
ST1568-SKR-ND
ST1518-COL-ND
ST2370-GER-BL, ST361-UNK-ND
ST1429-IRL-OS
ST1287-PRT-ND
ST64-USA-BL
ST2055-CHN-BL
ST1160-UNK-ND
ST1890-CHN-OS
ST1323-UNK-ND, ST1136-USA-ND
ST2842-CHN-ORO
ST2094-IRN-CA, ST3169-BRA-BL
ST1810-UK-ND
ST1683-IRL-OS, ST2315-KWT-BL
ST1824-IND-BL
ST2829-CHN-ORO
ST2274-UK-ND
ST735-ARG-BL
ST869-BEL-OS
ST1078-UK-ND
ST3191-UK-ND
ST2609-CHN-GM
ST1063-FRA-ND, ST2209-BRA-AN-AMM
ST2084-NOR-BL
ST2943-NZL-OS
ST1103-USA-AN-SQ
ST1391-MOR-VSW
ST2050-CHN-BL
ST3132-CHN-VSW
ST1929-CHN-OS
ST807-UK-BL
ST431-UK-BL
ST2518-UK-ND
ST2861-CHN-CA
ST1905-CHN-OS
ST359-BEL-OS
ST2611-CHN-ORO
ST111-BEL-AN-GO
ST1628-SKR-ND
ST2530-UK-ND
ST631-UK-ND
ST483-UK-VSW
ST2018-SKR-OS
ST1421-USA-ND
ST1165-BRA-ND, ST1426-IRL-OS, ST1184-BRA-ND
ST201-UK-BL
ST1205-UK-ND
ST1449-UK-ND
ST1887-CHN-VSW
ST1553-SKR-ND
ST2526-UK-ND
ST1825-UNK-ND
ST2910-TWN-BL
ST1290-PRT-ND
ST529-FRA-U, ST630-UK-ND, ST524-UK-BL
ST1860-CHN-SP
ST14-UNK-ND
ST2303-KWT-RS
ST1079-UK-ND
ST2299-KWT-BL
ST1017-UK-ND
ST1708-TWN-ND, ST3085-FRA-ND
ST182-MDG-VSW
ST3056-KWT-ND
ST818-JPN-BL
ST3142-CHN-VSW
ST1217-AUS-ORO
ST3058-IND-VSW
ST2059-CHN-BL
ST681-TWN-U
ST1756-TWN-ND
ST716-CHN-OS
ST71-USA-VSW
ST657-SAF-BL
ST2407-CHN-ML
ST2432-UK-ND
ST567-UK-BL
ST2951-CHN-VSW
ST3170-BRA-BL
ST2790-CHN-ORO
ST3027-SKR-OS
ST377-UK-OS
ST1714-TWN-ND
ST1758-TWN-ND, ST2774-CHN-ORO
ST2137-AUT-ND
ST203-UK-BL
ST3038-FRA-BL
ST1219-AUS-ORO
ST313-UK-BL
ST1874-CHN-VSW
ST2359-TWN-BL
ST1359-BRA-VSW
ST8-FRA-BL
ST2570-GER-BL
ST1623-SKR-ND
ST2865-UK-ND
ST2939-FRA-ND
ST1315-NOR-ORO
ST695-TWN-U
ST2279-FRA-ND
ST1289-PRT-ND
ST1685-IRL-OS
ST2681-CHN-ORO
ST1424-USA-ND
ST1489-FIN-ML
ST2868-CHN-ML
ST2413-CHN-BL
ST574-COL-W
ST2434-CHN-U
ST83-UK-BL
ST337-UK-BL
ST459-CHN-BL
ST103-UK-OS
ST2194-FRA-U

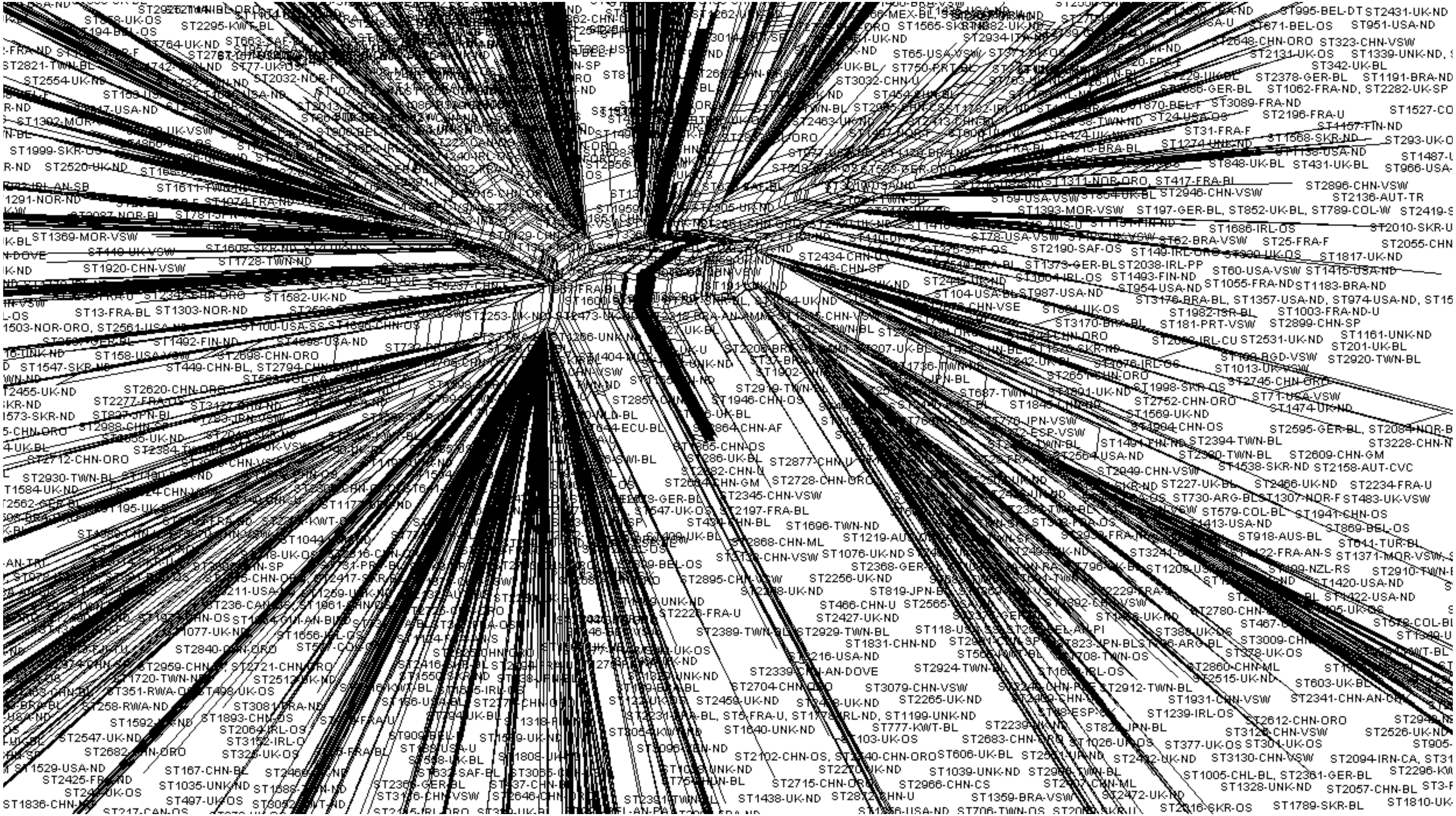

ST995-BEL-DT
ST2431-UK-ND
ST951-USA-ND
ST671-BEL-OS
ST2648-CHN-ORO
ST323-CHN-VSW
ST342-UK-BL
ST2378-GER-BL
ST1191-BRA-ND
ST1062-FRA-ND, ST2282-UK-SP
ST3089-FRA-ND
ST2196-FRA-U
ST1527-CO
ST1457-FIN-ND
ST31-FRA-F
ST1568-SKR-ND
ST848-UK-BL
ST431-UK-BL
ST2896-CHN-VSW
ST2136-AUT-TR
ST2946-CHN-VSW
ST197-GER-BL, ST852-UK-BL, ST789-COL-W
ST2010-SKR-U
ST1686-IRL-OS
ST62-BRA-VSW
ST25-FRA-F
ST2055-CHN
ST1817-UK-ND
ST2190-SAF-OS
ST1373-GER-BL
ST2038-IRL-PP
ST60-USA-VSW
ST1415-USA-ND
ST1055-FRA-ND
ST1183-BRA-ND
ST1493-FIN-ND
ST954-USA-ND
ST104-USA-BL
ST987-USA-ND
ST1982-ISR-BL
ST1003-FRA-ND-U
ST181-PRT-VSW
ST2899-CHN-SP
ST2531-UK-ND
ST1161-UNK-ND
ST201-UK-BL
ST1013-UK-VSW
ST2920-TWN-BL
ST2745-CHN-ORO
ST1998-SKR-OS
ST71-USA-VSW
ST2752-CHN-ORO
ST1474-UK-ND
ST1569-UK-ND
ST2595-GER-BL
ST3228-CHN-N
ST2394-TWN-BL
ST2609-CHN-GM
ST2158-AUT-CVC
ST1538-SKR-ND
ST2466-UK-ND
ST2234-FRA-U
ST483-UK-VSW
ST579-COL-BL
ST1941-CHN-OS
ST869-BEL-OS
ST918-AUS-BL
ST1420-USA-ND
ST1422-USA-ND
ST3009-CHN
ST378-UK-OS
ST603-UK-BL
ST2341-CHN-AN-CH
ST1931-CHN-VSW
ST1239-IRL-OS
ST2612-CHN-ORO
ST377-UK-OS
ST301-UK-OS
ST3130-CHN-VSW
ST1005-CHL-BL, ST2361-GER-BL
ST1328-UNK-ND
ST2057-CHN-BL
ST1789-SKR-BL
ST2016-SKR-OS
ST1810-UK
ST1836-CHN-ND
ST1529-USA-ND
ST2425-FRA-ND
ST497-UK-OS
ST1035-UNK-ND
ST167-CHN-BL
ST2682-CHN-ORO
ST2547-UK-ND
ST1592-UK-ND
ST258-RWA-ND
ST351-RWA-OS
ST498-UK-OS
ST1720-TWN-ND
ST2959-CHN-SP
ST2721-CHN-ORO
ST2840-CHN-ORO
ST1077-UK-ND
ST1656-IRL-OS
ST1893-CHN-OS
ST2064-IRL-OS
ST3081-FRA-ND
ST2512-UK-ND
ST1438-UK-ND
ST2715-CHN-ORO
ST2966-CHN-CS
ST1359-BRA-VSW
ST706-TWN-OS
ST1039-UNK-ND
ST2102-CHN-OS
ST606-UK-BL
ST2683-CHN-ORO
ST777-KWT-BL
ST1640-UNK-ND
ST2459-UK-ND
ST2265-UK-ND
ST3079-CHN-VSW
ST2924-TWN-BL
ST2339-CHN-AN-DOVE
ST216-USA-ND
ST1831-CHN-ND
ST2929-TWN-BL
ST2389-TWN-BL
ST2427-UK-ND
ST466-CHN-U
ST2256-UK-ND
ST1076-UK-ND
ST1219-AUS
ST1696-TWN-ND
ST2868-CHN-ML
ST2197-FRA-BL
ST2345-CHN-VSW
ST2728-CHN-ORO
ST2877-CHN-U
ST2864-CHN-AF
ST1946-CHN-OS
ST2919-TWN-BL
ST2434-CHN-U
ST2226-FRA-U
ST2895-CHN-VSW
ST2912-TWN-BL
ST1708-TWN-OS
ST2564-USA-ND
ST2949-CHN-VSW
ST227-UK-BL
ST730-ARG-BL
ST1307-NOR-F
ST2930-TWN-BL
ST1584-UK-ND
ST2712-CHN-ORO
ST2988-CHN-SP
ST2620-CHN-ORO
ST2277-FRA-OS
ST2455-UK-ND
ST1547-SKR-ND
ST158-USA-VSW
ST449-CHN-BL
ST2698-CHN-ORO
ST1492-FIN-ND
ST13-FRA-BL
ST1303-NOR-ND
ST1582-UK-ND
ST1920-CHN-VSW
ST1728-TWN-ND
ST1369-MOR-VSW
ST1999-SKR-OS
ST2520-UK-ND
ST2554-UK-ND
ST2821-TWN-BL
ST2295-KWT-BL
ST194-BEL-OS
ST2032-NOR-F
ST1565-SKR-ND
ST65-USA-VSW
ST750-PRT-BL
ST3032-CHN-U
ST2934-ITA-ND
ST59-USA-VSW
ST1393-MOR-VSW
ST1311-NOR-ORO, ST417-FRA-BL
ST3176-BRA-BL, ST1357-USA-ND, ST974-USA-ND
ST1491-FIN-ND
ST2980-TWN-BL
ST1845-CHN-ND
